# Characterization of human lightness discrimination thresholds for independent spectral variations

**DOI:** 10.1177/20416695241274662

**Published:** 2024-09-26

**Authors:** Devin Reynolds, Vijay Singh

**Affiliations:** Department of Physics, 3616 North Carolina Agricultural and Technical State University, Greensboro, North Carolina, United States

**Keywords:** lightness, human psychophysics, color vision, equivalent noise, spectral variations

## Abstract

The lightness of an object is an intrinsic property that depends on its surface reflectance spectrum. The visual system estimates an object's lightness from the light reflected off its surface. However, the reflected light also depends on object extrinsic properties of the scene, such as the light source. For stable perception, the visual system needs to discount the variations due to the object extrinsic properties. We characterize this perceptual stability for variation in two spectral properties of the scene: the reflectance spectra of background objects and the intensity of light sources. We measure human observers’ thresholds of discriminating computer-generated images of 3D scenes based on the lightness of a spherical target object in the scene. We measured change in discrimination thresholds as we varied the reflectance spectra of the objects and the intensity of the light sources in the scene, both individually and simultaneously. For small amounts of extrinsic variations, the discrimination thresholds remained nearly constant indicating that the thresholds were dominated by observers’ intrinsic representation of lightness. As extrinsic variation increased, it started affecting observers’ lightness judgment and the thresholds increased. We estimated that the effects of extrinsic variations were comparable to observers’ intrinsic variation in the representation of object lightness. Moreover, for simultaneous variation of these spectral properties, the increase in threshold squared compared to the no-variation condition was a linear sum of the corresponding increase in threshold squared for the individual properties, indicating that the variations from these independent sources combine linearly.

Our visual system provides perceptual representations of distal properties of objects based on the proximal stimuli captured by the eyes. While object properties are intrinsic to the object (its color, shape, etc.), the proximal stimuli also depend on the properties of the scene in which the object lies (object-extrinsic properties such as background objects in the scene, illumination, etc.) as well as the position and pose of the observer. The task of the visual system is to provide stable correlates of object-intrinsic properties under variability of the proximal signal due to object-extrinsic scene properties. This work quantifies the extent to which the visual system provides such stability for the representation of the reflectance of an object under variation in spectral properties of the scene, specifically, variation in the spectra of the background objects and the intensity of light sources in the scene.

The perceptual correlate of the diffuse spectral reflectance of an object is its perceived color. For achromatic objects, the analogous perceptual quantity is object lightness. The human visual system is known to provide a relatively stable representation of the color/lightness of an object despite variability in the proximal signal due to changes in the light source, the surface reflectance of other objects in the scene, and the geometry and other properties of the scene (Brainard & Radonjic, 2004; Foster, 2011). The degree to which such stability can be achieved is termed *color/lightness constancy* (Adelson, 2000; Gilchrist, 2006). Human color/lightness constancy has been measured using appearance-based approaches and discrimination-based approaches (Olkkonen & Ekroll, 2016). Appearance-based approaches involve tasks in which the observer makes a judgment about the appearance of stimuli. This approach includes methods such as color matching, color naming, scaling, and nulling (Foster, 2003). In color matching, observers adjust a test stimulus to match a standard stimulus. Color matching experiments show varying degrees of constancy, with constancy measured between 15% and 90% under conditions such as changes of illumination (Arend & Goldstein, 1987; Arend & Spehar, 1993), reflectance (Arend & Spehar, 1993; Patel et al., 2018), illumination gradients (Arend & Goldstein, 1990; Brainard et al., 1997), and illumination and simulated reflectance (Rutherford & Brainard, 2002). Color naming is a more direct and arguably natural method to measure color constancy where observers are asked to categorize stimuli based on their color (Troost & De Weert, 1991). This method has been used with real (Olkkonen et al., 2010; Uchikawa et al., 1989) and simulated stimuli (Olkkonen et al., 2009) to measure constancy. Color naming methods have the limitation that there is a vast number of possible discernible colors (Linhares et al., 2008), but typically observers are asked to name from a small set of color names (Hansen et al., 2007; Smithson & Zaidi, 2004; Speigle & Brainard, 1996) which might provide an overestimate of the measured constancy. In color scaling methods, observers view a stimulus and provide a rating on a scale for a set of colors, thus allowing for a finer level of comparison for measuring constancy (Luo et al., 1991; Schultz et al., 2006). Scaling methods can also be used to measure changes in stimuli, where observers provide a rating of the change between stimuli (Ennis & Doerschner, 2019). Nulling or achromatic adjustment methods involve changing a test stimulus such that it appears achromatic (Arend, 1993; Brainard, 1998; Delahunt & Brainard, 2004). This method has the limitation that it provides data only for achromatic/gray stimuli, and additional assumptions about the observers’ criterion need to be made for color appearances (Speigle & Brainard, 1996).

Discrimination-based approaches provide an objective method to measure color constancy (Bramwell & Hurlbert, 1996; Reeves et al., 2008). In these experiments, observers discriminate stimuli to be the same or different from each other. The stimuli are varied in some relevant parameter space to measure the threshold for discriminating changes in the parameter (Aston et al., 2019; Craven & Foster, 1992; Pearce et al., 2014). This approach was first developed to relate internal and external noise in the study of contrast sensitivity (Legge et al., 1987; Lu & Dosher, 1999). In the context of color constancy, discrimination-based approaches have been operationalized to relate discrimination thresholds as a measure of constancy (Craven & Foster, 1992). A high threshold for discriminating the change implies that the visual system can compensate for the effects of the change and hence has large perceptual constancy under the change. On the other hand, low discrimination thresholds imply poor constancy (Pearce et al., 2014). Using this approach in illumination discrimination tasks with a variety of illumination changes, it has been observed that the degree of constancy depends on both the starting illumination chromaticity and the chromatic direction (Aston et al., 2019; Pearce et al., 2014; Radonjić et al., 2018).

Recently, Singh et al. (2022) employed a discrimination-based approach in a lightness discrimination task to relate discrimination thresholds to the amount of extrinsic variation. They developed an *equivalent noise* paradigm to relate discrimination thresholds to the variability in observers’ intrinsic representation of object properties (e.g., its lightness) and to the variability in object-extrinsic properties of the scene. This approach, previously used for contrast sensitivity (Lu & Dosher, 1999), allowed them to relate task relevant and task irrelevant variations. They related discrimination thresholds to the variance in observers’ internal perceptual representation of lightness and the variance in object extrinsic properties. A comparison of these variances provides the degree of constancy in the object-intrinsic properties under variability in object-extrinsic properties.

The equivalent noise paradigm can also be used to compare different types of extrinsic variations and to characterize the combined effect of multiple extrinsic properties. In this work, we use the equivalent noise paradigm to compare how the variation in two spectral properties—background object reflectance and light source intensity—affect human lightness perception. We measure human observers’ threshold of discriminating two images based on the lightness of an achromatic target object as the variation in the reflectance spectra of background objects and the variation in the intensity of light source increases. Thresholds are measured for individual and simultaneous variations of these properties. Using the equivalent noise paradigm, the thresholds are related to the intrinsic and extrinsic variances, allowing the comparison of the effect of the spectral variations presented individually and simultaneously.

We show that as the variation in extrinsic sources increase, initially the thresholds remain nearly constant because the perceptual representation is dominated by intrinsic variability. As the extrinsic variability increases, it affects the perceptual representation, and the discrimination thresholds increase. Using a model based on signal detection theory, we show that the effect of extrinsic variation is within a factor of two compared to intrinsic variability. This indicates that the visual system provides a large degree of lightness constancy under object extrinsic scene variations. Comparing the thresholds of individual and simultaneous variations, we show that the effects of individual extrinsic variations combine linearly under simultaneous variation.

## Experimental Methods

### Overview

We followed the methods of a previous work (Singh et al., 2022) that studied the variation of reflectance spectra on lightness discrimination thresholds but used different stimuli. This section outlines the methods, emphasizing the changes compared to the previous work.

**Figure 1.**
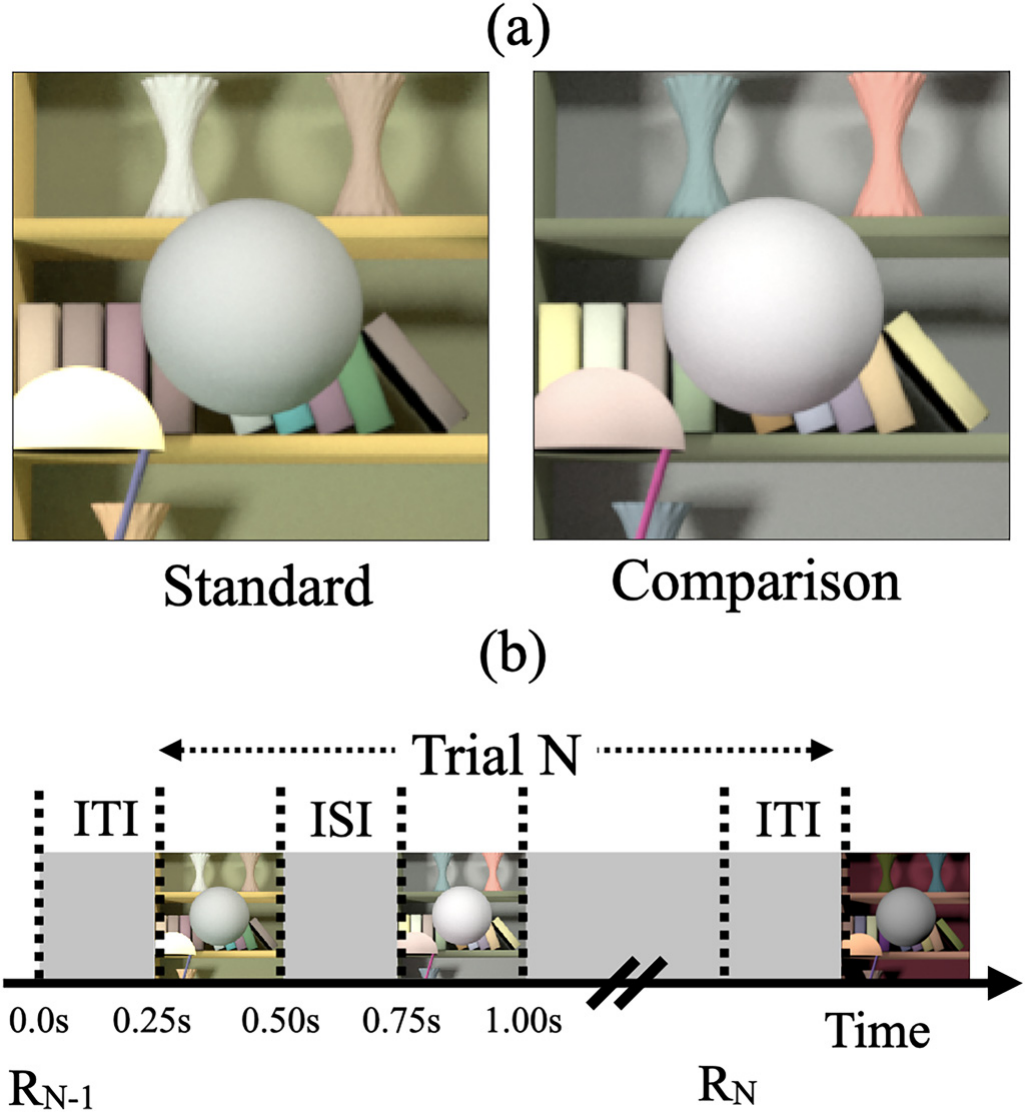
(a) Psychophysical task: The psychophysical task involved comparing two images on each trial, a standard image and a comparison image, and selecting the image with the lighter target object. In this panel, the spherical target object in the comparison image is lighter. We measured the threshold of discriminating the two images based on the lightness of the target object (Figure 2) and studied how these thresholds change as the trial-to-trial variability in the reflectance spectra of the background objects and the trial-to-trial variability in the intensity of the light sources increased. (b) Trial sequence: R*_N_*_−1_ indicates the recording of the observer's response for the (*N*−1)th trial. The *N*th trial begins 250 ms after the completion of the (*N*−1)th trial (inter trial interval, ITI = 250 ms). In the *N*th trial, the standard and comparison images are presented for 250 ms each with a 250 ms inter-stimulus interval (ISI) in between the two images. The order of the standard and comparison images is chosen in a pseudorandom order. The observer records their choice by pressing a button on a gamepad after both images have been presented and removed from the screen. The observers could take as long as they wish before making their choice. The recording of their choice is indicated by R*_N_* in the panel. The next trial begins 250 ms after the choice has been recorded. Adapted from Figure 1 in Singh et al. (2022).

**Figure 2.**
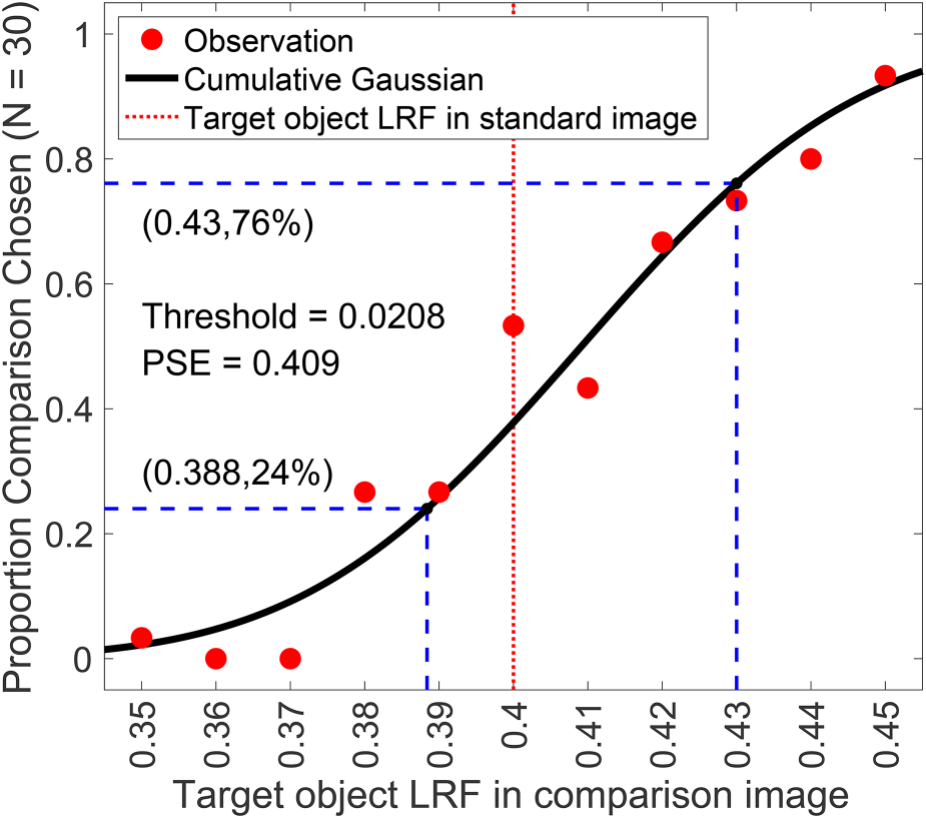
Psychometric function: We measured the proportion of times the observers selected the target in the comparison image to be lighter as a function of the LRF (luminous reflectance factor) of the target object. We collected 30 responses for each of the 11 equally spaced values of the comparison image target object LRF, ranging from 0.35 to 0.45. The LRF of the target object in the standard image was 0.40. We used maximum likelihood methods to fit a cumulative normal function to the proportion-comparison-chosen data. The guess rate and lapse rate were constrained to be equal and within the range 0 to 0.05. The threshold was defined as the difference between the LRF values corresponding to a proportion-comparison-chosen of 0.76 and 0.50, obtained from the cumulative normal fit. The figure presented here illustrates the data for observer 0003 in the second block of the *background reflectance variation* experiment for the no-variation ( $\sigma^{2}=0.00,\;\delta =0.00$ ) condition. The discrimination threshold was measured to be 0.0208. The point of subjective equality (PSE), which corresponds to a proportion of 0.5 in the comparison task, was found to be 0.409. The lapse rate for this fit was 0.00.

We used a two-alternative forced-choice (2AFC) procedure to measure thresholds (Figure 1). On each trial, observers viewed pairs of images of computer-generated 3D scenes on a color-calibrated monitor. These pairs consisted of a standard image and a comparison image and contained a centrally located achromatic sphere as the target object. Observers indicated the image in which the target object was lighter. Between trials, we manipulated the luminous reflectance factor (LRF) of the target object in the comparison image. The LRF is defined as the ratio of the luminance of a surface under a reference illuminant to the luminance of the reference illuminant itself (American Society for Testing and Materials, 2017). We chose the reference illuminant as CIE D65 standard illuminant. We plotted the psychometric functions of the observers by collecting data on the proportion of times the observer chose the comparison image to be lighter (see Figure 2). We fit this data with a cumulative normal function to estimate observers’ discrimination thresholds. We defined the threshold ( T ) as the difference between the target object LRF for which the cumulative normal fit equaled 0.76 and 0.50. This corresponds to a d-prime of 1 in a two-interval task.

We performed three preregistered experiments (see below Preregistration) to study the effect of two types of object-extrinsic spectral variations on human lightness discrimination thresholds:

1. *Background reflectance variation*: In this experiment, we measured human lightness discrimination thresholds as a function of the amount of variation in the background objects with fixed light source spectra.
2. *Light source intensity variation*: In this experiment, we measured human lightness discrimination thresholds as a function of the amount of variation in the intensity of light sources with a fixed background.
3. *Simultaneous variation*: In this experiment, we measured human lightness discrimination with simultaneous variation in the background object reflectance spectra and the light source intensity.

The samples of background object surface reflectance spectra were generated from a multivariate normal distribution modeled on databases of natural surface reflectance measurements. The amount of variation in the surface reflectance of background objects was varied by changing the size of the covariance matrix of the multivariate normal distribution. We measured discrimination thresholds for both chromatic and achromatic variations of background objects.

The shape of the light source spectral power distribution function was fixed, and its intensity was varied by multiplying the spectral power distribution function by a scalar sampled from a log uniform distribution. The amount of variation was controlled by changing the range of the log uniform distribution.

### Ethics Statement

All experiments were approved by North Carolina Agricultural and Technical State University Institutional Review Board and were in accordance with the World Medical Association Declaration of Helsinki.

### Preregistration

The experiments were preregistered before data collection. The preregistration documents are available at: https://osf.io/7tgy8/.^1^ The experiments were preregistered as Experiment 6 (referred to as *background reflectance variation*), Experiment 7 (referred to as *light source intensity variation*), and Experiment 8 (referred to as *simultaneous variation*). Experiment 6 replicated previous work (preregistered as Experiment 3; Singh et al., 2022) with additional achromatic background conditions. The experimental methods to measure lightness discrimination thresholds were the same for the three experiments.

The preregistration documents mentioned that the experiments aimed at characterizing the dependence of human lightness discrimination thresholds on the amount of variation in the background reflectance and the intensity of the light source in the scene. They described the method to estimate discrimination thresholds and predicted that the thresholds would increase with an increase in the amount of variation. We predicted that the thresholds of achromatic background variation would be lower than chromatic variation and the threshold for simultaneous variation would be higher than the threshold for individual variations. We also predicted that an equivalent noise model (Singh et al., 2022) would capture the increase in thresholds.

### Reflectance and Illumination Spectra

We used a statistical model of natural reflectance datasets to generate reflectance spectra of background objects (Singh et al., 2018, 2022). Two datasets, one containing 170 spectral measurements (Vrhel et al., 1994) and another containing 462 surface measurements (Kelly et al., 1943), were combined and mean-centered by subtracting out the mean surface reflectance of the 632 measurements. Then principal component analysis (PCA) was used to obtain the projection of the mean-centered dataset along the eigenvectors associated with the six largest eigenvalues. These eigenvalues captured more than 99.5% of the variance (Singh et al., 2018). The empirical distribution of the projection weights was approximated with a multivariate normal distribution. Pseudorandom samples were generated from this multivariate normal distribution to obtain the projection weights of the samples of reflectance spectra. Reflectance spectra were constructed by using these projection weights along with the eigenvectors and adding the mean of the surface reflectance dataset. A physical realizability condition was imposed on these spectra by ensuring that the reflectance at each wavelength was between 0 and 1. If a reflectance spectrum did not meet this criterion, it was discarded.

For achromatic spectra, after generating a physically realizable reflectance spectrum, its average reflectance over all wavelengths was calculated and it was replaced by a spectrum that had this average reflectance at all wavelengths.

The amount of spectral variation was controlled by multiplying the covariance matrix of the multivariate normal distribution by a covariance scalar ( $\sigma^{2}$ ). A covariance scalar of 0 indicates no variation in the reflectance spectra of the background objects and a covariance scalar of 1 corresponds to variations observed in the natural reflectance datasets.

The light source power spectrum was chosen to be the CIE D65 reference illuminant. The D65 spectrum was divided by its mean power over wavelength to obtain its relative spectral shape. Variation in the light source intensity was introduced by multiplying the normalized D65 spectrum by a random number generated from a log-uniform distribution in the interval [1− δ , 1+ δ ], where δ determines the range. We chose a log-uniform distribution for the multiplication parameter because the spectral power distribution functions of natural daylight spectra vary over three orders of magnitude and their mean across wavelength can be roughly approximated by a log-uniform distribution (Singh et al., 2018). All light sources in a scene were assigned the same power spectrum.

**Figure 3.**
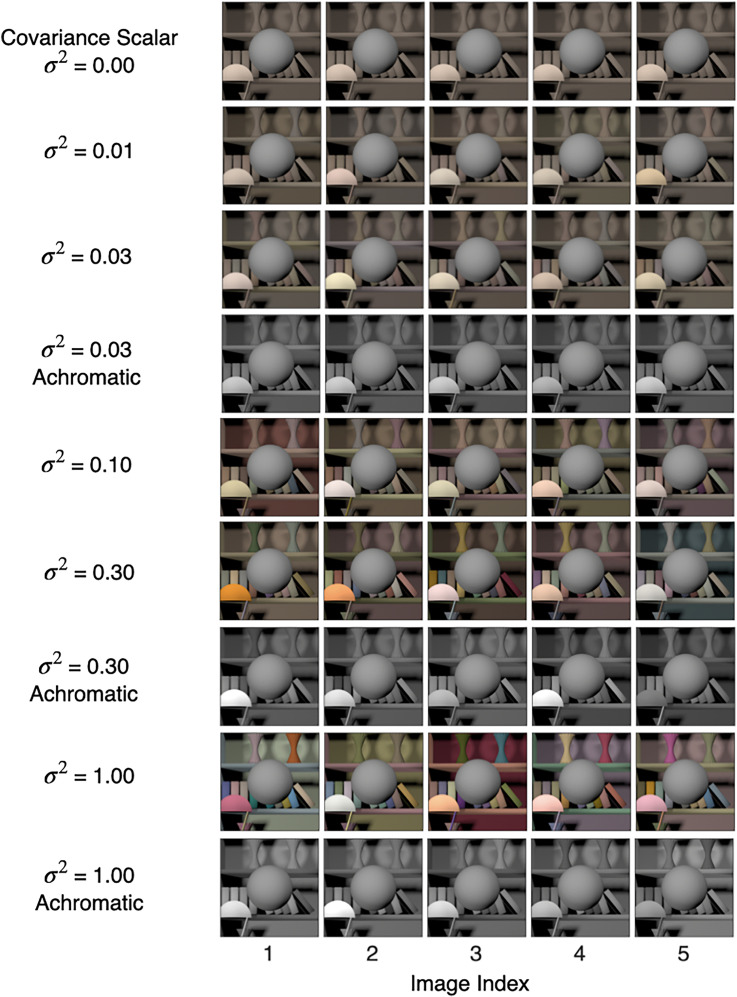
Background reflectance variation: We studied both chromatic and achromatic variation in the reflectance spectra of background objects. For each condition, we generated 1100 images—100 images at each of the 11 linearly spaced values of the target object luminous reflectance factor (LRF) in the range [0.35, 0.45]. The figure shows five typical images for each of these nine conditions, indicated by the *x*-label. The target object in each image in the figure is at LRF = 0.4. The images at the LRF level 0.4 were used as both standard and comparison images.

**Figure 4.**
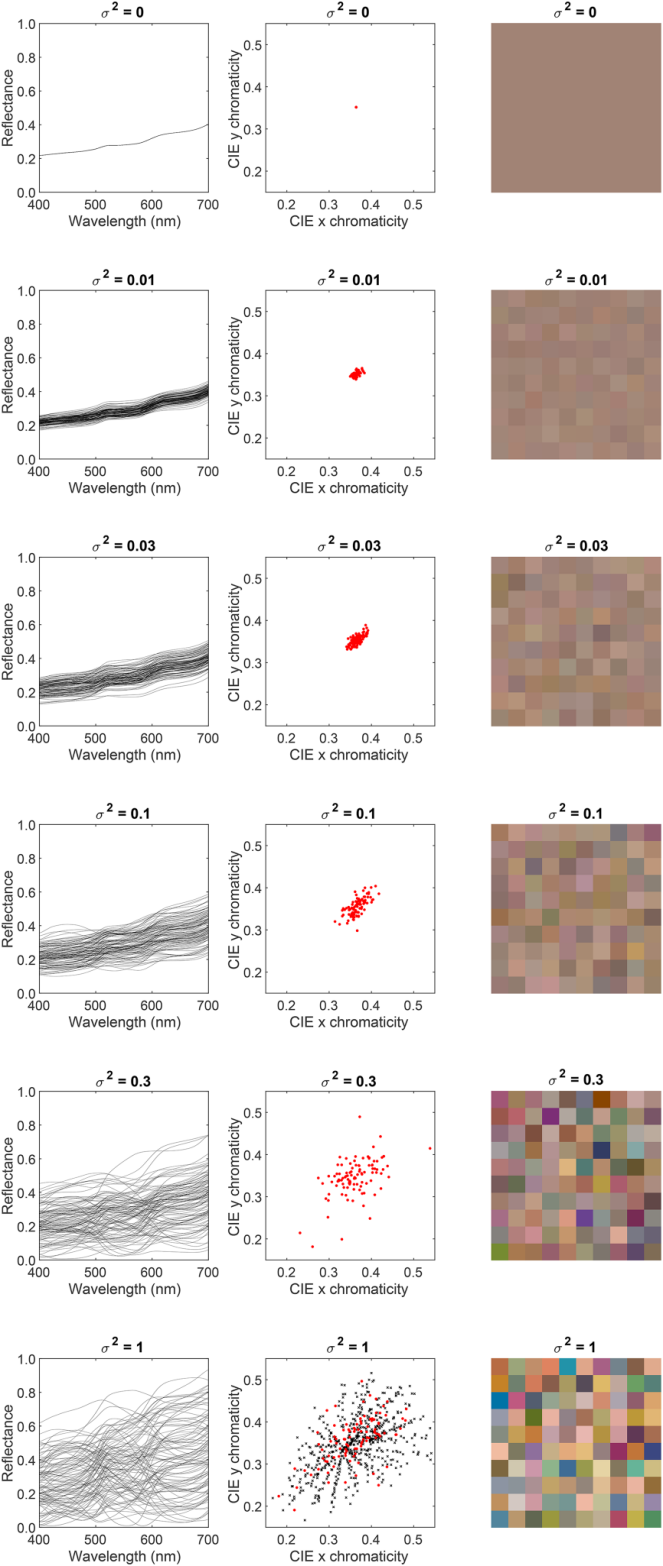
Statistical model of surface reflectance: (Left column) Samples of the statistical model of background object reflectance spectra. The panels show 100 samples for the six covariance scalars. (Middle column) The CIE xy chromaticity of the samples is shown in the left column. The bottom row also shows the CIE xy chromaticity of the 632 surface reflectance measurements on which the model is based (black “x” markers). (Right column) sRGB renditions of the samples are shown in the left column. (See Figure S1 in the online supplemental materials for achromatic condition.)

The values of the two parameters $\sigma^{2}$ and δ for the three experiments were as follows:

*Background reflectance variation*: In this experiment, we generated images for nine conditions. Six of these conditions were for chromatic variation at six logarithmically spaced values of the covariance scalar ( $\sigma^{2}$ ): [0, 0.01, 0.03, 0.1, 0.3, 1.0] (same as Singh et al., 2022). Three conditions were for achromatic variation at the covariance scalar ( $\sigma^{2}$ ): [0.03, 0.3, 1.0]. The power spectrum of the light source was the same for all images. Figure 3 shows five typical images for the nine conditions. The relative spectra, CIE xy chromaticity, and the color renditions of the typical reflectance spectra used in the chromatic conditions of the *Background reflectance variation* experiment are shown in Figure 4 (see Figure S1 in the online supplemental materials for the achromatic condition). When the value of the covariance scalar is zero, the relative spectrum is equal to the mean of the combined surface reflectance measurements (Kelly et al., 1943; Vrhel et al., 1994); the color rendition is grayish with CIE x and y chromaticity equal to 0.364 and 0.352, respectively. As the value of the covariance scalar increases, the variation in the relative shape of the reflectance spectra increases. The model captures the statistical properties of the surface reflectance dataset (Singh et al., 2018).

**Figure 5.**
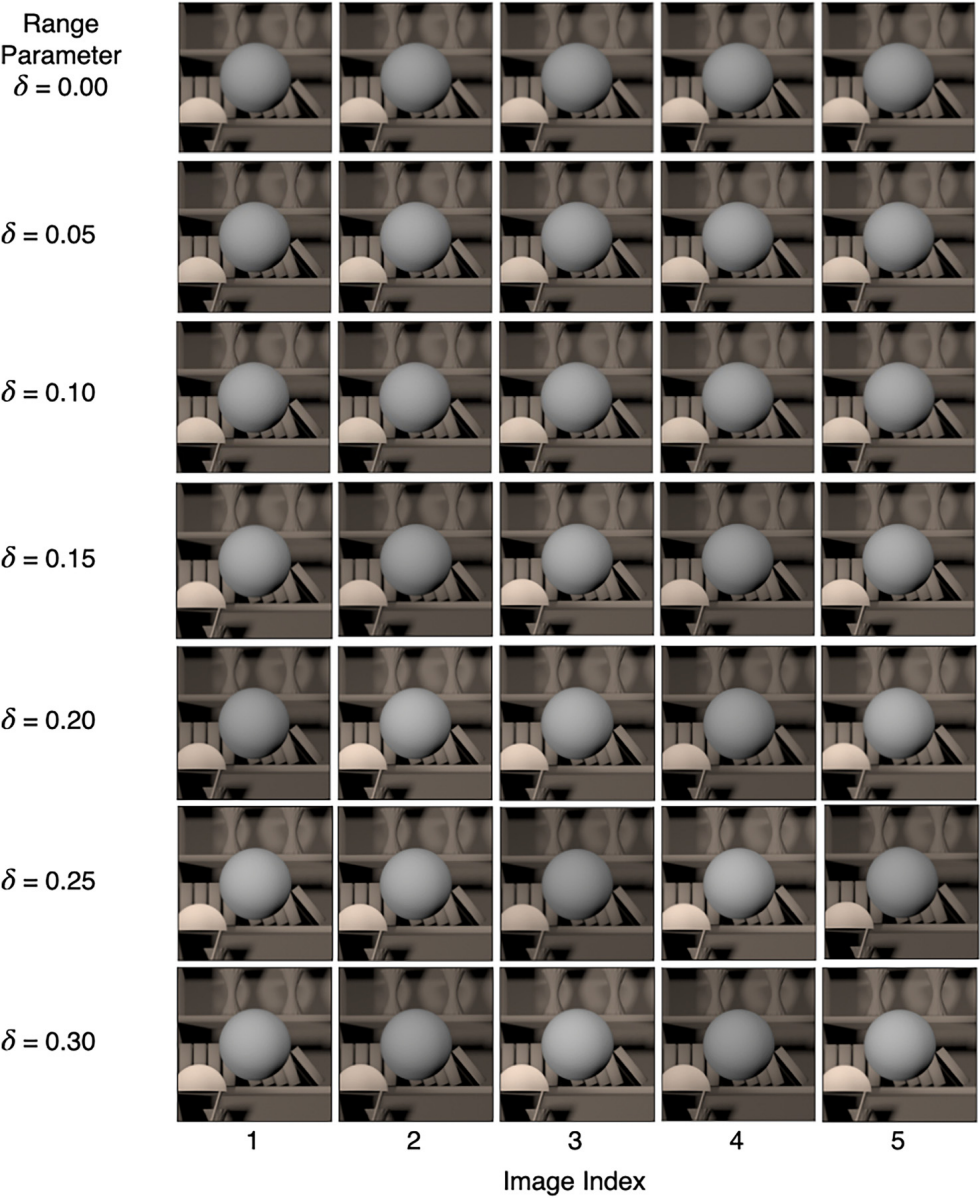
Light intensity variation: We measured light intensity variation at seven linearly spaced values of the range parameter in the range [0.00, 0.30]. For each value, we generated 1100 images, 100 images at each value of the target object luminous reflectance factor (LRF) in the range [0.35, 0.45]. The figure shows five sample images for each value of the range parameter. The target object in each image in the figure has the same LRF of 0.40.

**Figure 6.**
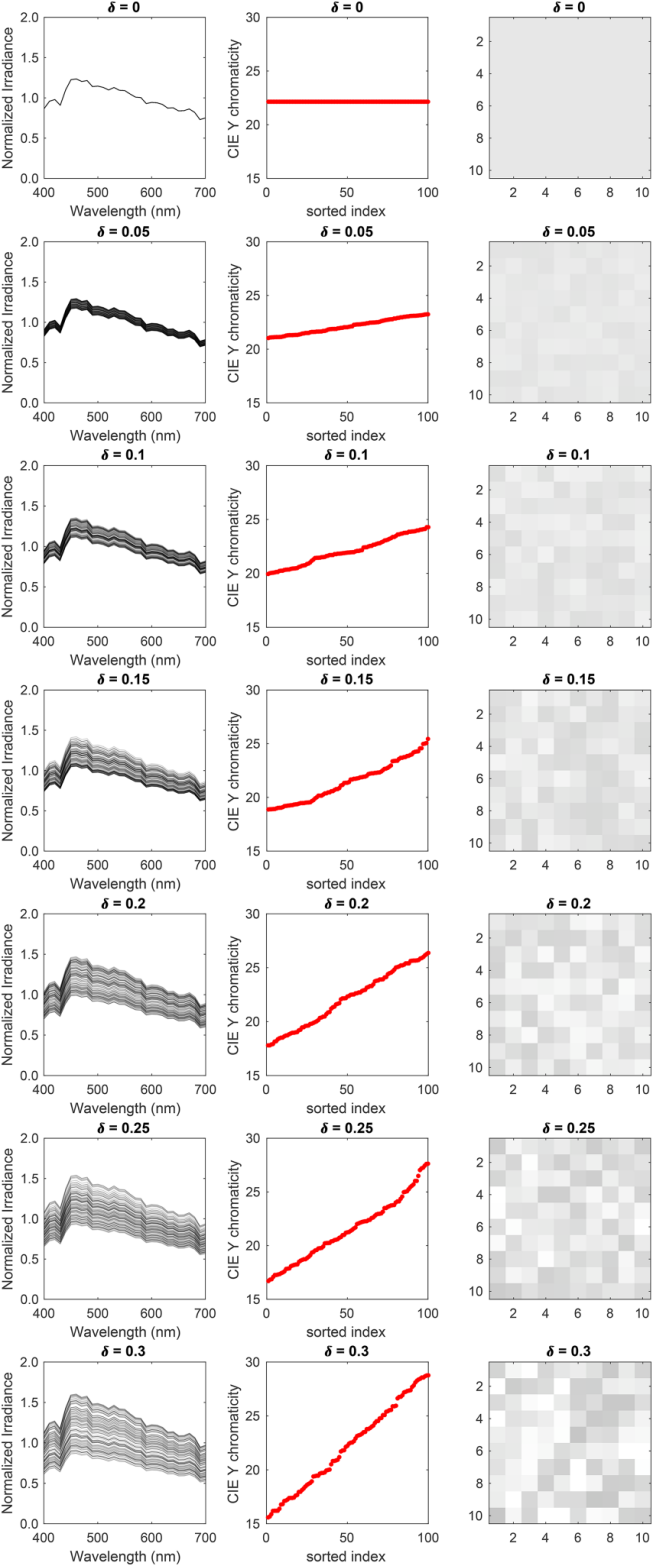
Statistical model of illumination spectra: (Left column) Samples of the statistical model of the light source power spectra. Each panel shows 100 samples for the seven values of the range parameter. (Middle column) CIE Y chromaticity of the samples is shown in the left column. We have sorted the chromaticity in an increasing order to illustrate the range over which the chromaticity varies. (Right column) sRGB renditions of the samples in the left column.

*Light source intensity variation*: In this experiment, we generated images for seven linearly spaced values of the range parameter ( δ ): [0.00, 0.05, 0.10, 0.15, 0.20, 0.25, 0.30]. The reflectance spectra of all background objects were the same and were equal to the mean spectrum of the reflectance database. This corresponds to a covariance scalar of 0. Figure 5 shows five typical images for the seven conditions. As the range parameter increases, one can notice the difference in the luminance of the individual panels. Figure 6 shows the relative spectra (left column), CIE Y chromaticity (middle column), and the rendered color renditions (right column) of 100 random draws of the light source power spectrum for the seven range parameters. Due to the fixed shape of the power spectrum (normalized D65 spectrum), their CIE x and y chromaticity are the same, equal to 0.313 and 0.329, respectively. The variation is in the relative value, which increases with an increase in the range parameter, leading to higher variability in the CIE Y chromaticity.

**Figure 7.**
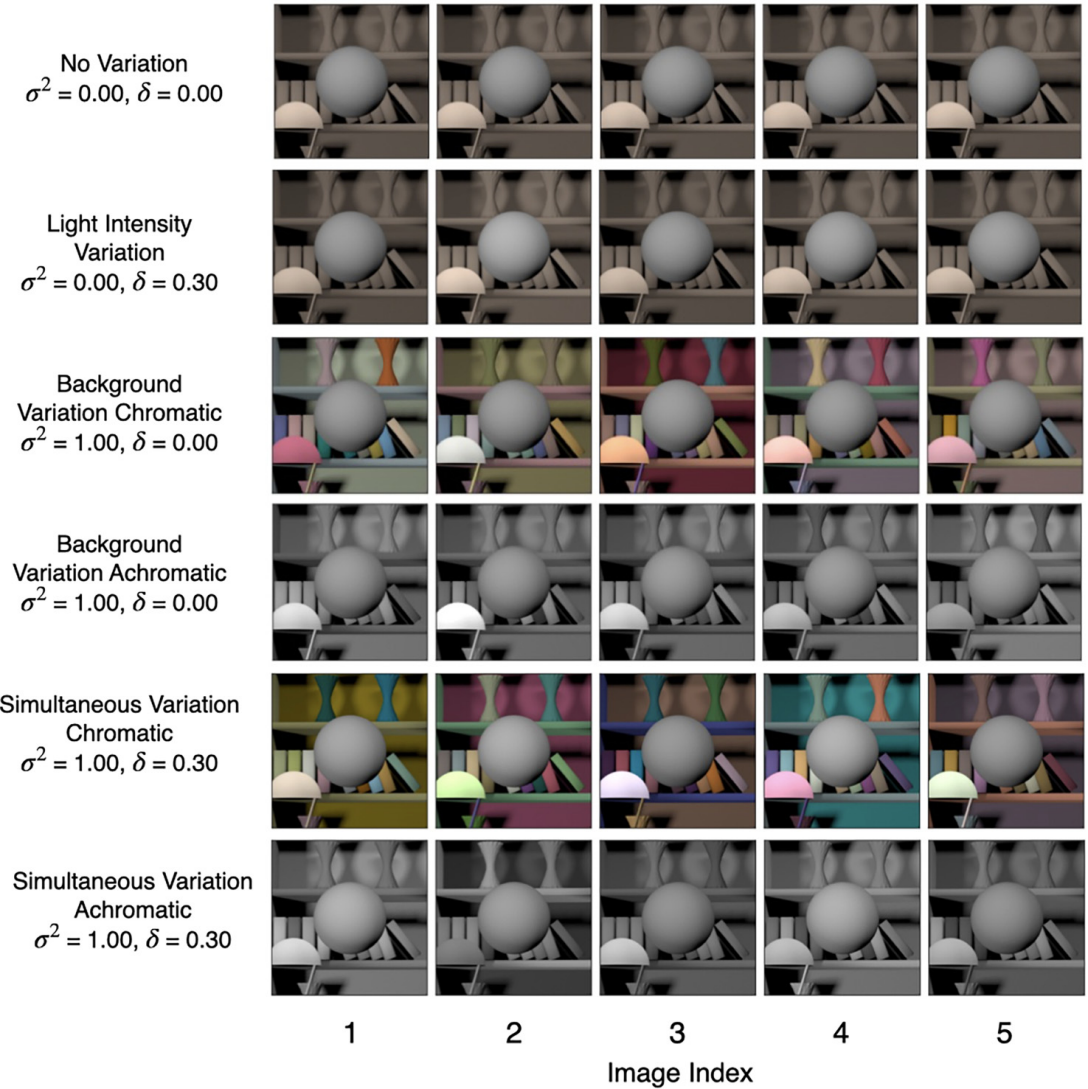
Simultaneous variation: This figure shows five sample images for the six conditions studied in the *simultaneous variation* experiment. We generated 1100 images for each of these conditions—100 images at each value of the target object luminous reflectance factor (LRF) in the range [0.35, 0.45].

*Simultaneous variation*: In this experiment, we studied six conditions. These were: no-variation ( $\sigma^{2}=0$ , δ=0 ), chromatic background variation ( $\sigma^{2}=1$ , δ=0 ), achromatic background variation ( $\sigma^{2}=1$ , δ=0 ), light source intensity variation ( $\sigma^{2}=0$ , δ=0.3 ), simultaneous variation chromatic background ( $\sigma^{2}=1$ , δ=0.3 ), and simultaneous variation achromatic background ( $\sigma^{2}=1$ , δ=0.3 ). Figure 7 shows five typical images for these six conditions.

### Image Generation

The images used in this work were generated using the software Virtual World Color Constancy (VWCC) (github.com/BrainardLab/VirtualWorldColorConstancy) as described in (Singh et al., 2018). The software package produces physically accurate models of computational visual scenes. The rationale for using a rendering package is that it allows us to construct images where the key properties of the scene are under programmatic control. The package also allows the use of statistical models of spectral properties of the scene, such as the object reflectance function or the light source spectral power function. Thus, one can generate large-scale, well-labeled datasets with specific types of variations while keeping other properties of the scene fixed.

The initial step to generate an image involves constructing a 3D model that serves as the base scene. Additional objects and light sources can be inserted in the base scene for a richer 3D geometry. As this work focused on the effects of spectral properties of the scene and not the geometrical aspects, we fixed the 3D geometry for all images. We followed the methods in Singh et al. (Singh et al., 2018) to generate images. For the 3D model, we chose the *Library* base scene from the base scenes available in VWCC and additionally inserted a spherical object and a spherical light source. The spherical object was chosen as the target object. This choice was made to avoid geometrical effects such as self-shadowing. The target object was a distance 11.4 m from the camera. The *Library* base scene contained two additional area lights. The next step was to assign reflectance spectra and spectral power distribution functions to the objects and light sources within the base scene based on the specific experimental condition for which the images were generated. While rendering an image, all light sources within the scene were assigned the same spectral power distribution function. All surfaces in the scene were chosen to be matte and had no specularity. The images did not include secondary reflections to avoid any variability in the images due to such reflection. Previously, it has been shown that secondary reflections have a negligible effect on lightness discrimination thresholds (Singh et al., 2022).

Next, we inserted a camera in the scene, pointed it to the center of the inserted spherical object, and rendered a 2D multispectral image of the scene using Mitsuba (version 0.5.0), a physically-realistic open-source renderer (mitsuba-renderer.org; Jakob, 2010). The image was rendered at 31 wavelengths in the range 400–700 nm spaced 10 nm apart. As observed from the camera position in the *Library* base scene, the image corresponded to a 17° field of view and was 320-pixel by 240-pixel in size. A 201-pixel by 201-pixel part of the image centered at the target object was cropped out to display on the monitor. Figure 1a shows two instances of the cropped image. The multispectral images were first converted into LMS images using Stockman–Sharpe 2° cone fundamentals, then into RGB images using monitor calibration data. A common scaling was applied to ensure that all images used in an experiment were within the monitor gamut. The gamma corrected RGB images were presented on the calibrated monitor (Singh et al., 2022).

### Stimulus Design

For each condition described above, we generated 1100 images, 100 images at each of the 11 linearly spaced values of the target object LRF in the range [0.35, 0.45]. The standard image target object LRF was 0.4. The comparison image target object LRF varied in the range [0.35, 0.45]. We generated 100 images at each comparison level to avoid excessive replication of images in the experiment. The images at LRF level 0.4 were used as both standard and comparison images. For the no-variation condition ( $\sigma^{2}$ = 0.00, δ = 0.00), we generated one image at each target object LRF level, as the background reflectance and the light source intensity remained fixed in this case.

The standard image, when presented on the experimental monitor, had an average luminance of 87.1cd/m^2^ for the no-variation condition ( $\sigma^{2}$ = 0.00, δ = 0.00). The average luminance of the target object for the 11 LRF levels were 120.9, 122.3, 123.8, 125.2, 126.5, 127.9, 129.2, 130.5, 131.9, 133.1, and 134.4 cd/m^2^. For the simultaneous background and light source intensity variation condition ( $\sigma^{2}$ = 1.00, δ = 0.30), the average luminance of the standard image was 87.8 cd/m^2^. The average luminance of the target object for the 11 LRF levels were 117.7, 119.4, 119.4, 122.3, 123.7, 123.8, 127.8, 126.9, 127.7, 129.1, and 129.0 cd/m^2^.

### Experimental Structure

In this study, a trial is defined as displaying a standard and a comparison image on the monitor and recording the observer's response. An interval is defined as the presentation of either the standard image or the comparison image within a trial. A block consists of recording 330 trials for one condition, and 30 trials at each of the 11 comparison image target LRF levels. A permutation involved recording one block of data for each condition in an experiment. We recorded three permutations for each observer in each experiment. Each permutation had a random order of the blocks.

The order of the blocks in a permutation, the LRF levels of the comparison image in trials of a block, and the order of standard and comparison images in a trial were generated pseudorandomly and stored at the beginning of the experiment for each observer. The 30 comparison images at one LRF level of a block were chosen pseudorandomly with replacement from the 100 images available at that LRF level. Before starting a new permutation for an observer, the data for all blocks (conditions) in a permutation were collected.

A session consisted of recording three blocks on a single day. An observer performed no more than one session a day. Each block was divided into three sub-blocks of 110 trials, with the observers taking a minimum 1 min break between subblocks and a 2–5 min break between blocks. The observers were informed that they could stop the experiment at any time. If the observer terminated a block, the data were not recorded. No observer terminated a block of the experiment.

Each observer first performed a practice session in which three blocks of data were recorded for the no-variation ( $\sigma^{2}=0$ , δ=0 ) condition. At the beginning of the practice session, the experimenter explained the experimental procedure to the observer and obtained their consent. Vision tests were performed to ensure normal visual acuity and normal color vision. Then the observer was familiarized with the experimental setup by performing a familiarization block of 40 trials. Observers set their head on a headrest and fixated at a fixation dot on the monitor. They were instructed that the trial would start on the click of the gamepad. They would see two images appear and disappear in sequence with a blank screen between the images. They were asked to focus on the spherical object at the center of the images and indicate the image in which the central sphere was lighter using the gamepad. It was explained that the discrimination performed was between light versus dark.

After familiarization, the observer was dark adapted by sitting in the dark for about 5 min. Then the data for the three blocks of the practice session were recorded. At the end of the practice session, the observer was informed if they could continue the experiment based on their mean threshold. If the observer was continued, their data were collected over several sessions. The no-variation condition was also included in each permutation of the experiment. So, data for the no-variation condition were collected again. Data collection for the six observers took place over several weeks. To ensure that all images in an experiment were presented with the same monitor settings, the experimental monitor was calibrated before each experiment (see Monitor Calibration section).

The *simultaneous variation* experiment contained the individual variation blocks (background reflectance variation only and light source power spectrum variation only), as well as the simultaneous variation block (both background reflectance and light source power spectrum variation). These blocks were randomized for each observer (see above).

### Observer Recruitment and Exclusion

Observers were recruited from North Carolina Agricultural and Technical State University and the local community and were compensated for their time. The observers were screened for the normal visual acuity of 20/40 or better (with corrective eyewear if necessary) and normal color vision, which were assessed using pseudo-isochromatic plates (Ishihara, 1977).

We then conducted a practice session to identify observers who could reliably perform the psychophysical task. We first familiarized the observers with the experiment by asking them to respond to 40 trials of the experiment for the no-variation condition ( $\sigma^{2}=0.00$ , δ=0.00 ). These trials were divided into 10 easy trials, 10 moderate trials, and 20 regular trials. In easy trials, they compared images with target object LRF 0.35 to images with target object LRF 0.45. In moderate trials, they compared a standard image with target object LRF 0.40 to images with target object LRF either 0.35 or 0.45. In regular trials, they compared the standard image with target object LRF 0.40 to comparison images with target object LRF chosen randomly in the range [0.35, 0.45]. The data for these 40 trials were not stored. After familiarizing with the experiment, the observer performed three blocks of the experiment for the no-variation condition ( $\sigma^{2}=0.00$ , δ=0.00 ). The threshold was calculated for these three blocks. If the mean threshold of the observer for the last two blocks in the practice session was larger than 0.030, the observer was discontinued. The preregistration document specified these exclusion criteria (see Methods: Preregistration). If the observer met these criteria, they continued with the rest of the experiment.

For each observer, the practice session was performed at the beginning of each of the three experiments, irrespective of whether the observer had participated in an earlier experiment. Table 1 summarizes the demographic information of the observers in the three experiments.

**Table 1.** Information about the observers who participated in the three experiments.

Experiment	Background reflectance variation	Light source intensity variation	Simultaneous variation
Total	25	15	20
Male/female	15/10	6/9	11/9
Age range	19–34 (mean: 23.33)	19–33 (mean: 23.83)	19–28 (mean: 20.8)
Pseudo-names and visual acuity	*0003* (L20/30, R20/20)	*0003* (L20/30, R20/20)	*0003* (L20/30, R20/20)
	*bagel* (L20/20, R20/20)	*bagel* (L20/20, R20/20)	*bagel* (L20/20, R20/20)
	*committee*^a^ (L20/25, R20/25)	*oven*^b^ (L20/20, R20/20)	*oven* (L20/20, R20/20)
	*content*^a^ (L20/30, R20/20)	*content*^a^ (L20/30, R20/20)	*content*^a^ (L20/30, R20/20)
	*observer*^a^ (L20/25, R20/25)	*primary*^a^ (L20/20, R20/20)	*manos*^c^ (L20/25, R20/25)
	*revival* (L20/20, R20/20)	*revival* (L20/20, R20/20)	*revival*^c^ (L20/20, R20/20)

*Note.* Total: total number of observers who participated in the practice session of the experiment; male/female: total number of male and female participants; age range: age range of the participants; pseudo-names and visual acuity: pseudo-names and visual acuity of observers who passed screening. Visual acuities are provided in parentheses. These observers are a subset of the observers listed in the “Total” row.

a These observers used personal corrective eyewear during vision test and the experiments.

b Observer *oven* reported some difficulties during a few sessions of the light intensity variation experiment and their thresholds for two conditions did not fit the expected pattern. We removed their data from the analysis presented in this work. Their data and thresholds are provided in Table S2 in the online supplemental materials.

c Due to a lack of observers who met the preregistration criteria for the simultaneous variation experiment, two observers (manos and revival), whose thresholds were close to the preregistration criteria, were also retained for the experiment. Observer revival had participated in the previous two experiments and had met the criteria both times. Observer manos showed improvement in thresholds with each block, with the threshold for the final block below 0.03. This was a deviation from the preregistration.

### Apparatus

The experiments were performed in a dark room and the stimuli were presented on a color-calibrated LCD monitor (27-in. NEC MultiSync EA271U; NEC Display Solutions). The pixel resolution of the monitor was selected as 1920 × 1080. Its refresh rate was 60 Hz and each RGB channel was operated at 8-bit resolution. The experimental computer was an Apple Macintosh with an Intel Core i7 processor. The experimental programs were written in MATLAB (MathWorks; Natick, MA, USA) and utilized Psychophysics Toolbox (http://psychtoolbox.org) and mgl (http://justingardner.net/doku.php/mgl/overview) libraries. A Logitech F310 gamepad controller was used to collect observers’ responses.

The distance between the observers’ eyes and the monitor was set at 75 cm. A forehead rest and chin cup (Headspot, UHCOTech, Houston, TX, USA) were used to stabilize the observers’ head position. The observers’ eyes were centered both horizontally and vertically in relation to the display.

### Monitor Calibration

The monitor was calibrated using a spectroradiometer (PhotoResearch PR655) as described in (Singh et al., 2022). For this, we focused the spectroradiometer on a 4.66 cm × 4.66 cm (3.56°×3.56°) patch on the monitor. The radiometer sampled from a 1° circular spot at the center for the patch due to its optics. We measured the spectral power distribution of the three monitor primaries in the range 380–720 nm at 4 nm intervals. For each primary, the gamma function was determined by measuring the spectral power distribution at 26 equally spaced values in the range [0, 1] where 1 is the maximum allowed input and 0 is no input. The linearity of the monitor was also checked by measuring the spectral power distribution at 32 different combinations of the input in the range [0,0,0] and [1,1,1]. The maximum absolute deviation between measured and predicted values were 0.0087 and 0.0081 for x and y chromaticity, respectively. For luminance, the maximum absolute deviation was less than 1%.

The monitor was calibrated before starting each experiment. Once calibrated, the same settings were used until data for all observers for that experiment were collected. The monitor was then recalibrated for the next experiment. Data were collected in the sequence *background reflectance variation* experiment, *light source intensity variation* experiment, and *simultaneous variation* experiment.

### Stimulus Presentation

The images were presented on a color calibrated monitor. When displayed on the experimental monitor, the size of the image was 2.6 cm × 2.6 cm corresponding to a 2° × 2° visual angle for the observer located at 75 cm from the monitor. The size of the spherical target object was ∼1°. This stimulus size was chosen considering the comparable size of receptive fields in the human visual cortex (Dumoulin & Wandell, 2008; Yoshor et al., 2007) and observations that human observers fixate on a small part of scenes during color judgments (Radonjić et al., 2016), with color awareness decreasing sharply away from the center of the field of view (Cohen et al., 2020). The rest of the monitor was set at the lowest value input [0,0,0]. Except for a 4 cm × 4 cm square part concentric to the image presentation location, the rest of the monitor was also covered with black paper. The images were presented for 250 ms each with a 250 ms inter-stimulus interval (ISI) between the images. The monitor was dark (lowest value input [0,0,0]) during the ISI. The observer's response was collected after both images were presented and removed from the monitor. The observer could take as long as they wished before responding. The observers were provided auditory feedback about correct or incorrect response after their response. The next trial started 250 ms after the feedback. Thus, the actual inter trial interval depended on the response time of the observer.

### Code and Data Availability

The data for each experiment and observer are provided as supplementary information (SI) available at: https://github.com/vijaysoophie/SimultaneousVariationPaper. The SI contains the proportion-comparison-chosen data as well as the thresholds for the three experimental blocks of each condition, for each experiment and observer. The MATLAB scripts used to generate Figures 2, 4, 6, and 8–14, and Figures S1–S8 in the online supplemental materials, and to obtain thresholds of the linear receptive field formulation of the model are also provided in the SI.

**Figure 8.**
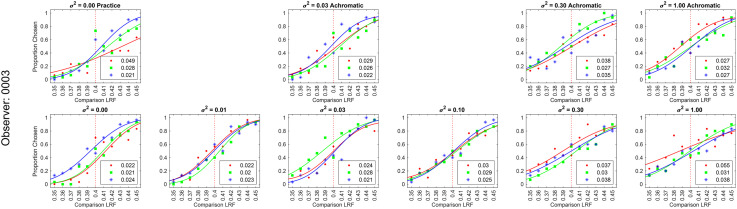
Psychometric functions of observer 0003 for background reflectance variation experiment: We measured the proportion-comparison-chosen data for the nine conditions separately in three blocks for each observer. The figure shows the psychometric function for observer 0003. The psychometric functions for all six observers are shown in Figure S3 in the online supplemental materials. A cumulative normal function was fit to the data from each block to determine the discrimination threshold (see Figure 2). The legend provides the estimated lightness discrimination threshold for each block, obtained from the cumulative fit. The first panel in the top row shows the data and thresholds for the practice session. An observer was selected for the experiment only if the average of their last two discrimination threshold measurements in the practice session was less than 0.30. The last three panels in the top row show the data for the three achromatic conditions. The bottom row shows the data for the chromatic variation conditions.

### Linear Receptive Field Model

The experiments characterize how the variance in task-irrelevant variables are related to lightness discrimination thresholds. To quantify the relationship between the variance and the discrimination thresholds, we fit the measured thresholds to a previously developed linear receptive field (LINRF) model (Singh et al., 2022), which is based on signal detection theory (Green & Swets, 1995).

In the standard formulation of signal detection theory for a 2AFC task, two identical Gaussian random variables are compared and the one with the higher value is selected as corresponding to the signal. The discriminability of the two variables is measured using a quantity called d-prime ( $d^{\prime }$ ). d-prime is the ratio of the difference between the mean of the two Gaussian random variables and the standard deviation of the distributions. Thus, d-prime measures the distance between the means of the two distributions in the units of the standard deviation. $d^{\prime }=0$ corresponds to the distributions being identical. Larger values of $d^{\prime }$ indicate higher discriminability between the two distributions.

In the LINRF model, the visual response to the target object is determined by the response of a linear receptive field to the image. Even for images with target objects at the same LRF, the visual response can vary because of two factors. One, due to object extrinsic properties such as the background reflectance or the light source power spectrum, which change from image to image. This is the extrinsic noise ( $\eta_{e}$ ). The second factor is the internal response variability in the visual system. This is the intrinsic noise ( $\eta_{i}$ ) and is modeled as an additive Gaussian perturbation. The model compares the visual response to the standard and comparison images and chooses the image with the higher visual response as the one with the lighter target object. Since the noise models used in the LINRF model are linear and Gaussian, signal detection theory can be used to relate the overall variance in the visual response to the discrimination threshold of the model as explained below.

The response of the receptive field is calculated as the dot product of a center surround receptive field (*R*) with the image (*I*). By representing *I* and *R* as vectors, where the elements of *I* represent the radiant power emitted by the monitor and the elements of *R* represent the receptive field sensitivity at the corresponding location, the visual response ( r ) is given as $r=R^{T}I+\eta_{i}$ , where $\eta_{i}$ is a mean zero Gaussian perturbation.

For the standard and the comparison images, the visual response can be written as:

(1) $$r_{is}=R^{T}(I_{s0}+\eta_{e})+\eta_{i}=R^{T}I_{s0}+\eta$$

(2) $$r_{ic}=R^{T}(I_{c0}+\eta_{e})+\eta_{i}=R^{T}I_{c0}+\eta$$

Here, $I_{s0}$ and $I_{c0}$ represent the standard and comparison images without noise, $\eta_{e}$ represents the extrinsic noise due to variations in the images, $\eta_{i}$ represents the intrinsic noise of the observer, and η the overall noise. Since the receptive field and noise models are assumed to be linear and Gaussian, the overall variance ( $\sigma_{\eta }^{2}$ ) can be related to the variance of the intrinsic noise ( $\sigma_{i}^{2}$ ) and the covariance matrix of the extrinsic noise ( $\Sigma_{e}$ ) as:

(3) $$\sigma_{\eta }^{2}=(\sigma_{i}^{2}\;+R^{T}\Sigma_{e}R)$$

Thus, the visual response to the standard and the comparison images have a Gaussian distribution around the mean $R^{T}I_{s0}$ and $R^{T}I_{c0}.$ Using signal detection theory, the difference between the mean receptive field response to the standard and the comparison image, $R^{T}(I_{s0}-I_{c0})$ , can be related to the standard deviation of the overall variability ( $\sigma_{\eta }$ ) using the proportionality constant d-prime ( $d^{\prime }$ ).

(4) $$R^{T}(I_{s0}-I_{c0})=d^{\prime }\sigma_{\eta }$$

We assume that the mean difference $R^{T}(I_{s0}-I_{c0})$ is proportional to the difference between the LRF level of the target object in the standard and the comparison images ( $\Delta_{\mathrm{LRF}}$ ), i.e., $R^{T}(I_{s0}-I_{c0})\;\;\propto \;\Delta_{\mathrm{LRF}}$ . The proportionality constant can be absorbed in the definition of the receptive field response, essentially measuring the receptive field response in the units of the LRF, leading to $R^{T}(I_{s0}-I_{c0})\;=\Delta_{\mathrm{LRF}}.\;$ Thus, we get:

(5) $$\Delta_{\mathrm{LRF}}=d^{\prime }\;\sigma_{\eta }$$

When the LRF of the target object in the standard and comparison images are the same, assuming there is no bias, the proportion comparison chosen will be equal to 0.50. In our experiment, the threshold (*T*) is defined as the difference between the LRF of the target object in the comparison images for which the proportion comparison chosen are equal to 0.76 and 0.50. This choice of the threshold corresponds to the condition where the difference between the mean response to the standard image and the comparison image is equal to the variance, leading to $d^{\prime }=1$ (Green & Swets, 1995). Thus, for $\Delta_{\mathrm{LRF}}=T$ , the thresholds (*T*) can be related to the extrinsic and intrinsic noise as:

(6) $$T=\sqrt{\;\sigma_{i}^{2}\;+R^{T}\Sigma_{e}R}\;\mathrm{or}\;T^{2}=\sigma_{i}^{2}\;+R^{T}\Sigma_{e}R$$

In the *background reflectance variation experiment*, the extrinsic noise is varied by multiplying the covariance matrix of the multinormal distribution from which reflectance spectra are sampled with a covariance scalar (see Reflectance and Illumination Spectra). Thus, we can write $R^{T}\Sigma_{e}R=\sigma^{2}\times R^{T}\Sigma_{e0}R$ , where $\Sigma_{e0}$ is the covariance matrix of the extrinsic noise corresponding to the variation in the dataset of natural images and $\sigma^{2}$ is the covariance scalar. Thus, the model relates the threshold-squared ( $T^{2}$ ) in the experiments to the variance in the intrinsic noise of the observer ( $\sigma_{i}^{2}$ ) and the variance in the extrinsic noise $(\sigma_{e0}^{2}=R^{T}\Sigma_{e0}R$ ) through the relation:

(7) $$T^{2}=\sigma_{i}^{2}+\sigma^{2}\times \sigma_{e0}^{2}$$

In our experiments, we chose $\sigma^{2}$ on a logarithmic scale. So, we have represented the data using the form:

(8) $$\log\;T^{2}=\log\;(\sigma_{i}^{2}+\sigma^{2}\times \sigma_{e0}^{2})$$

### Fitting the LINRF Model Using a Simulation Approach

We fit the LINRF model to the threshold data with a simulation approach. Following the approach of Singh et al. (Singh et al., 2018), we first generate the response of a model visual system to the images used as stimuli in the experiment. For this, we simulated the isomerization of a model retinal cone mosaic using the software ISETBio (Cottaris et al., 2019). The retinal mosaic had a total of 2601 cones with long (L), middle (M) and short (S) cones in the ratio L:M:S = 0.60:0.30:0.10 (1523 L-cones, 801 M-cones, and 277 S-cones). The model also included optical blur, axial aberration, and chromatic aberration. Cone isomerizations were simulated for a 100 ms integration time, incorporating Poisson noise. The isomerizations were demosaiced using linear interpolation to estimate the L, M, and S cone isomerization images. The isomerization images were normalized by the quantal efficiency (integrated over the wavelength) of each cone class to ensure comparable response magnitudes. The normalized cone isomerization images were used as the input stimuli (*I*) for the LINRF model.

The receptive field response (*r*) to the image was calculated by taking the dot product of a center-surround receptive field (*R*) with the LMS cone response image (*I*). The receptive field was square in shape to match the images, with a circular center corresponding to target object's size and location. The central region had a uniform positive sensitivity of 1, while the surround had a uniform negative sensitivity equal to $v_{s}$ . Three copies of the receptive field were used, one for each cone class. The receptive field response was the sum of the dot product of the receptive field with the three cone response images. A mean zero Gaussian noise was added to the sum.

The LINRF model threshold was calculated using the 2AFC paradigm as the psychophysics experiment. For each trial, we simulated the noise-added receptive field response for the standard and comparison images and chose the image with the higher response as having the lighter target object. We performed 10,000 trials at each of the 11 comparison target object LRF levels to simulate the proportion comparison chosen data. The data were fit to the psychometric function to obtain the model threshold. The two parameters of the model, the variance of the intrinsic noise ( $\sigma_{i}^{2}$ ), and the value of the receptive field surround sensitivity $\left(v_{s}\right)$ were chosen independently for the *background reflectance variation* experiment and the *light source intensity variation* experiment.

*Background reflectance variation:* In this case, the Gaussian noise variance ( $\sigma_{i}^{2}$ ) and the surround sensitivity $\left(v_{s}\right)$ were chosen to minimize the mean squared difference between the thresholds of the LINRF model and the experimental thresholds measured at six values of the covariance scalar. We calculated the threshold of the LINRF model for a range of values of the parameters $\sigma_{i}^{2}$ and $v_{s}$ . The mean squared difference between the model threshold and the experimental threshold were calculated as a function of the parameters $\sigma_{i}^{2}$ and $v_{s}$ and were fitted to a second-degree polynomial of two variables. Minimizing this polynomial provided the best-fit parameters, denoted as $\sigma_{i,B}^{2}$ and $v_{s,B}$ . (The subscript B has been introduced to distinguish the *background reflectance variation* experiment from the *light source intensity variation* experiment.) $\sigma_{i,B}^{2}$ provided the estimate of the intrinsic noise. The extrinsic noise ( $\sigma_{e0,B}^{2}$ ) was estimated by using the best-fit surround sensitivity ( $v_{s,B}$ ) and the sample covariance matrix of the images ( $\Sigma_{e0}$ ) at $\sigma^{2}$ = 1 in the relation $R^{T}\Sigma_{e0}R$ .

*Light source intensity variation:* In this case, we fit the experimental thresholds to a functional form similar to Equation (7) where we replace the covariance scalar $\sigma^{2}$ by the range parameter δ . We minimized the mean squared difference between the experimental and LINRF model thresholds measured for the seven values of the range parameter ( δ ), as explained above for the *background reflectance variation* experiment, to obtain the minimum values of the Gaussian noise variance ( $\sigma_{i}^{2}$ ) and receptive field surround sensitivity ( $v_{s}$ ). We denote these minimum parameters as $\sigma_{i,L}^{2}$ and $v_{s,L}$ . (The subscript L has been introduced to distinguish the *light source intensity variation* from the *background reflectance variation* experiment.) $\sigma_{i,L}^{2}$ provided the estimate of the observer's intrinsic noise.

The intensity variation in natural daylight spectra of several orders of magnitude (Singh et al., 2018) cannot be captured in the experiment due to limitations of the monitor. So, to estimate extrinsic noise variance, we used the best-fit surround sensitivity $v_{s,L}$ and the sample covariance matrices to calculate the quantity $R^{T}\Sigma_{e0}R$ as a function of the range parameter δ . We fit the resulting values with an exponential function (see Figure S2 in the online supplemental materials) and chose the extrinsic noise variance ( $\sigma_{e0,L}^{2}$ ) as the value of the exponential fit at δ=1 . The parameter ( $\sigma_{e0,L}^{2}$ ) could be used to estimate the extrinsic noise variance for more naturalistic variations using the exponential fit.

## Results

### Human Lightness Discrimination Thresholds Increase with Background Reflectance Variation

We measured lightness discrimination thresholds of six human observers for both chromatic and achromatic variation in the surface reflectance of background objects in the scene. We measured thresholds for six chromatic condition and three achromatic conditions (see Methods Reflectance and Illumination Spectra). For each observer and condition, discrimination thresholds were measured three times in three separate blocks. The psychometric functions of one observer are shown in Figure 8 (see Figure S3 in the online supplemental materials for all six observers). The trend in psychometric functions is as expected. As the covariance scalar increases, the slope of the psychometric functions decreases, corresponding to an increase in discrimination thresholds. This trend is consistent for chromatic and achromatic conditions, as well as, across observers.

**Figure 9.**
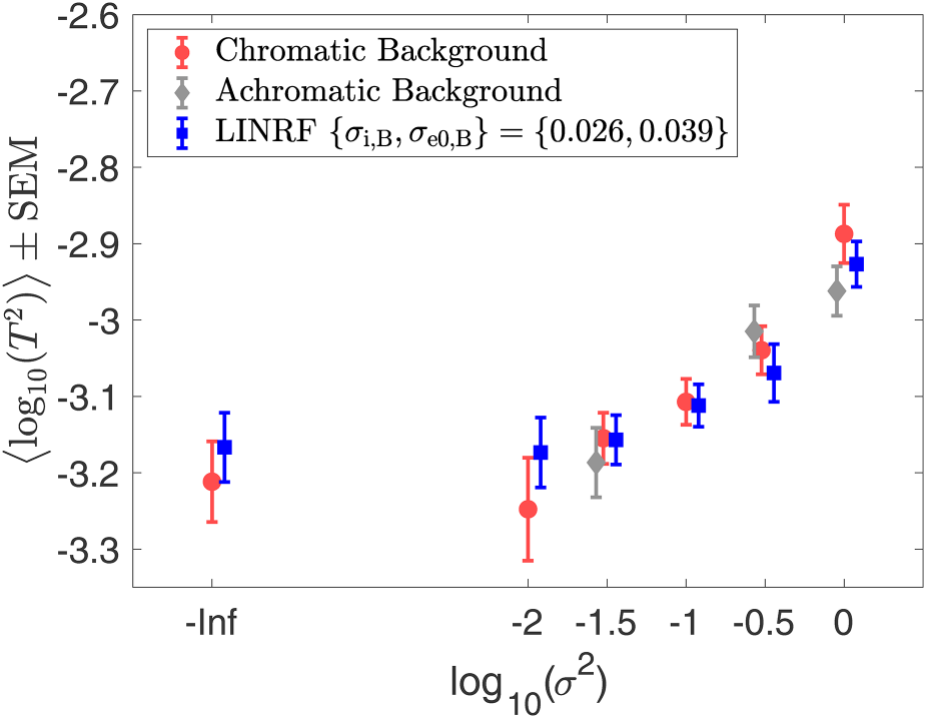
Background reflectance variation increases lightness discrimination thresholds: Mean (*N* = 6) log squared threshold vs. log covariance scalar from human psychophysics for chromatic (red circles) and achromatic conditions (gray diamonds). The error bars represent ±1 SEM taken between observers. The threshold of the linear receptive field (LINRF) model was estimated by simulation for the six values of the covariance scalar (blue squares). The blue error bars show ±1 standard deviation estimated over 10 independent estimates of the LINRF model parameters. The legend shows the parameters of the linear receptive field (LINRF) model fit. The data have been jittered for ease of viewing. A comparison of the thresholds with the previously published data in Singh et al., 2022 is shown in Figure S4 in the online supplemental materials.

Figure 9 shows the discrimination thresholds as a function of the variance in the reflectance of the background objects. We plot the mean log threshold squared (averaged across observers, *N* = 6) as a function of the log of the covariance scalar. The thresholds and standard error of the mean (SEM) from Figure 9 are listed in Table S1 in the online supplemental materials. We observe that at small values of the covariance scalar, the thresholds are nearly constant. As the value of the covariance scalar increases, log threshold squared also increases. The thresholds are comparable for chromatic and achromatic variation. We used one-way ANOVA to test the hypothesis that the mean thresholds for chromatic and achromatic variations are equal. The one-way ANOVA *p*-values were 0.72, 0.57, and 0.16 for the covariance scalar values 0.03, 0.30, and 1.00, respectively. This indicates that the differences in mean thresholds for chromatic and achromatic conditions are not statistically significant. Figure S4 in the online supplemental materials shows that the discrimination thresholds for the chromatic conditions are consistent with previously reported thresholds by Singh et al. (Singh et al., 2022).

We fit the thresholds to the LINRF model (Equation 7) developed by (Singh et al., 2022). The LINRF model provides an estimate of the variance of the internal noise of the observer as $\sigma_{i,B}^{2}=0.026$ and the variance of the extrinsic variability due to the reflectance of background objects as $\sigma_{e0,B}^{2}=0.039$ . The equivalent noise level, the ratio of the external variance to intrinsic noise, is ∼ 1.5, indicating that the variability in the representation of object lightness induced by the natural variability in the reflectance of background objects is close to the internal variability of that representation. If the ratio was equal to 1, we would have concluded that the visual system has discounted the external variability. However, the ratio is not significantly large compared to 1, indicating that there is significant lightness constancy.

### Human Lightness Discrimination Thresholds Increase with Light Source Intensity Variation

**Figure 10.**
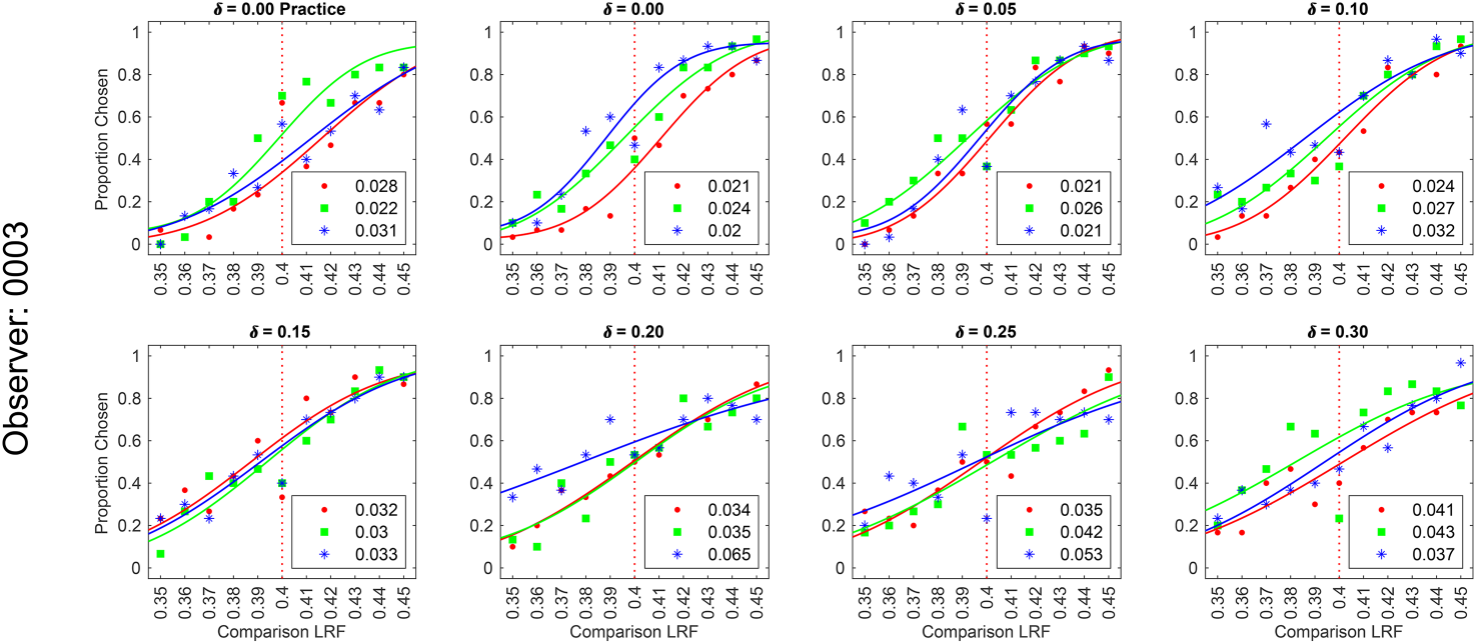
Psychometric functions for observer 0003 for the light source intensity variation experiment: Same as Figure 8, but for the *light source intensity variation* experiment. The figure shows the proportion comparison chosen data for the selection session and the seven conditions for observer 0003. The legend provides the estimated lightness discrimination threshold for each block, obtained from the cumulative fit. The psychometric functions for all observers are shown in Figure S5 in the online supplemental materials.

We measured lightness discrimination thresholds of six human observers for seven levels of variation in the intensity of light sources in the scene. The psychometric functions of one of the observers for these seven conditions are shown in Figure 10 (see Figure S5 in the online supplemental materials for all six observers). As expected, as the range parameter increases, the psychometric functions become shallower, the slope of the curve decreases, indicating an increase in discrimination thresholds. This trend is consistent over multiple measurements and observers.

**Figure 11.**
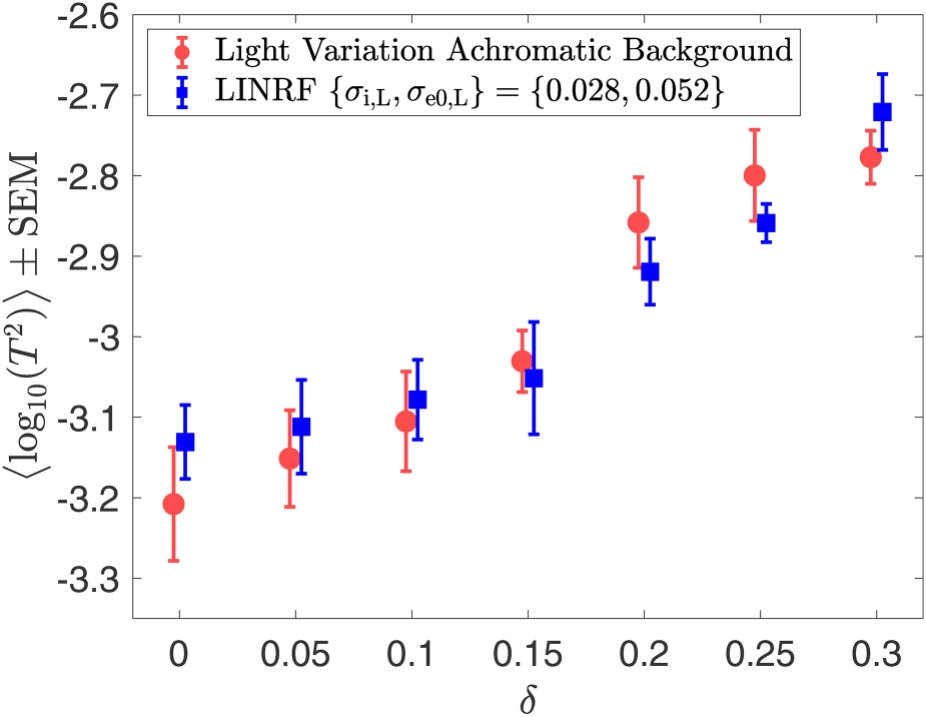
Light source intensity variation increases lightness discrimination threshold: Mean (*N* = 5) log squared threshold vs. range parameter from human psychophysics for the seven *light source intensity variation* conditions (red circles). The error bars represent ±1 SEM taken between observers. The threshold of the linear receptive field (LINRF) model was estimated by simulation for the seven values of the range parameters (blue squares). The blue error bars show ±1 standard deviation estimated over 10 independent estimates of the LINRF model parameters. The legend shows the parameters of the LINRF model fit. The data have been jittered for ease of viewing. The data for all six observers are shown in Figure S6 in the online supplemental materials.

Figure 11 shows the change in thresholds as the amount of variation in the light source intensity increases. The data is averaged over five observers (the data for one of the observers have been removed from Figure 11, see Figure S6 in the online supplemental materials for the data for all observers). Table S2 in the online supplemental materials lists the mean thresholds and the SEM measured in this experiment. Similar to the trend for reflectance spectra variation, lightness discrimination thresholds remain nearly constant for a small amount of variation, and then the log threshold squared increases as the amount of variation increases. A fit of the mean squared threshold with the LINRF model gives the value of internal noise as $\sigma_{i,L}^{2}=0.028$ . This compares well with the internal noise obtained from the *background reflectance variation* experiment ( $\sigma_{i,B}^{2}=0.026$ ). The variance of the extrinsic variability estimated at the range parameter δ=1.00 is $\sigma_{e0,L}^{2}=0.052$ . The equivalent noise level, the ratio of external variation to intrinsic noise is ∼1.8. This indicates that the variation in the lightness representation induced by the variation in light source intensity is close to the internal variation of that representation at these levels. In natural conditions, where light source intensity varies over several orders of magnitude, the extrinsic noise can be estimated by generating images at the level of variation and using the LINRF model.

### Thresholds for Simultaneous Variation are Higher Than Individual Variations

**Figure 12.**
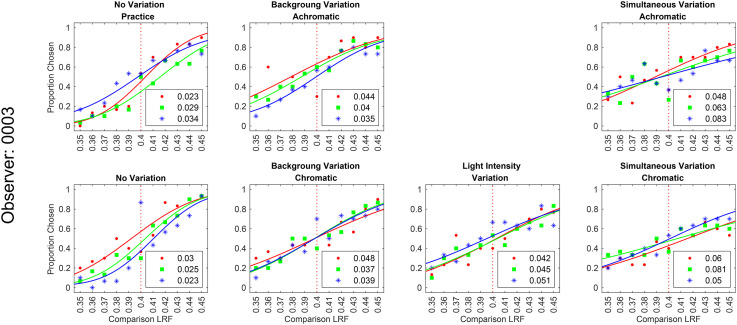
Psychometric functions for observer 0003 for simultaneous variation experiment: Same as Figures 8 and 10, but for the simultaneous variation experiment. The figure shows the proportion comparison chosen data for the selection session and the six conditions for observer 0003. The legend provides the estimated lightness discrimination threshold for each block, obtained from the cumulative fit. The data for all observers are shown in Figure S7 in the online supplemental materials.

**Figure 13.**
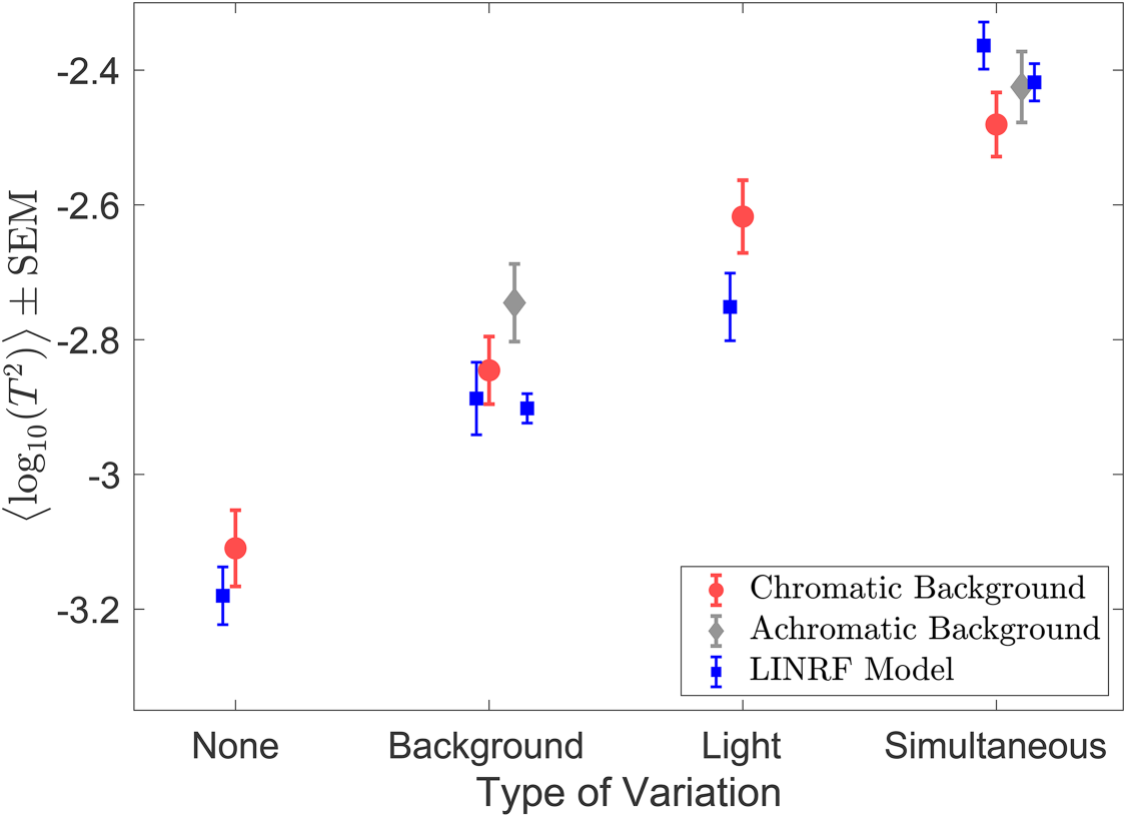
Discrimination thresholds for simultaneous variation of two sources are higher than individual discrimination thresholds: Mean (*N* = 6) log squared threshold for the six conditions in the simultaneous variation experiment. The error bars represent ±1 SEM taken between observers. The data for chromatic (red circles) and achromatic (gray diamonds) conditions have been plotted next to each other for visual comparison. The thresholds of the linear receptive field (LINRF) model (blue squares) were estimated using the parameters of the background variation condition (Figure 9) for the none, background variation, and simultaneous variation conditions and using the parameters of the *light intensity variation* experiment (Figure 11) for the light condition. The blue error bars show ±1 standard deviation estimated over 10 independent estimates of the LINRF model parameters. See Figure S8 in the online supplemental materials for LINRF model thresholds with the same set of parameters for all conditions.

We measured lightness discrimination thresholds for simultaneous variation in the reflectance spectra of background objects and the intensity of the light sources in the scene. In this experiment, we studied six conditions: no-variation, variation in the reflectance spectra of background objects with a fixed spectrum of the light sources for achromatic and chromatic backgrounds, variation in the intensity of light sources with a fixed background, and simultaneous variation in the intensity of the light sources and the reflectance spectra of background objects for chromatic and achromatic backgrounds. We measured the lightness discrimination thresholds of six human observers for these six conditions. The psychometric function of one of the observers is shown in Figure 12 (see Figure S7 in the online supplemental materials for all six observers). Figure 13 shows the mean log squared threshold of all six observers for these six conditions. Table S3 in the online supplemental materials lists the mean thresholds and SEM from Figure 13. The threshold for simultaneous variation of light source intensity and reflectance spectra of background objects is higher than the condition with individual variations in these properties. As observed earlier, the threshold for achromatic and chromatic conditions are comparable. We used one-way ANOVA to test the hypothesis that the mean thresholds for chromatic and achromatic variations are equal. The one-way ANOVA *p*-value is 0.19 for the background variation condition and 0.44 for the simultaneous variation condition. This indicates that the differences in the mean thresholds are not statistically significant.

Figure 13 also shows the squared thresholds of the LINRF model for the six conditions. We used the intrinsic noise and the surround sensitivity ( $v_{s}$ ) parameters of the *background reflectance variation* experiment to estimate the threshold of the LINRF model for the no-variation condition, background spectra variation conditions, and simultaneous variation conditions (Experiment 6, Figure 9). For the light intensity variation condition, we used the parameters of the *light source intensity variation* experiment (Experiment 7, Figure 11). See Figure S8 in the online supplemental materials for the predictions of the LINRF model with the same set of parameters for all six conditions.

We can use the LINRF model to compare the extrinsic variance of the simultaneous variation condition to the variance of the individual variations. According to the linear receptive model, the square of the threshold is proportional to the sum of the variance of observers’ intrinsic noise and the extrinsic variation in the stimuli (Equation 7). The squared threshold at the no-variation condition is equal to the variance of the observers’ intrinsic noise. In case of extrinsic variation, the increase in the threshold squared compared to the no-variation condition equals the variance of the extrinsic variation. When there is more than one independent source of extrinsic variation, the total variance of the simultaneous variation should be the sum of the variance of the individual variations. This predicts that the increase in threshold squared for simultaneous variation conditions should be equal to the sum of the corresponding increase for the individual variation conditions.

**Figure 14.**
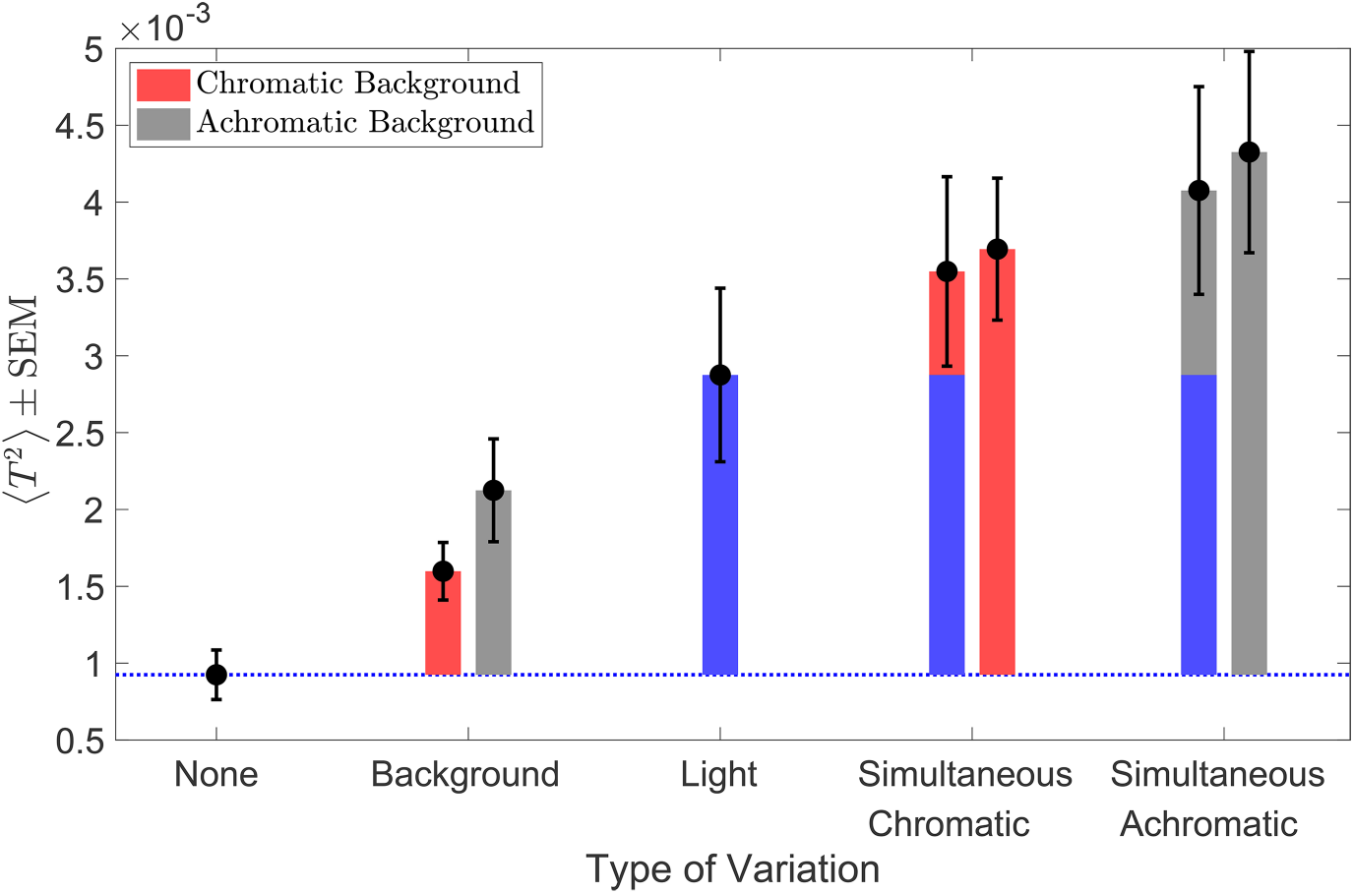
Extrinsic noise of independent variations adds linearly for simultaneous variation: Mean squared thresholds (*N* = 6) for the six conditions in the simultaneous variation experiment (black circles). The black error bars represent ±1 SEM taken between observers. The bars (red, gray, and blue) represent the increase in squared thresholds compared to the no-variation condition (blue dotted line). The red bar is for the chromatic background condition, the gray bar for achromatic background condition, and the blue bar for the light intensity variation condition. For the simultaneous variation conditions, the bars on the right (bars with one color—red or gray) represent the increase in the measured squared threshold and the bars on the left (stacked bars of two different colors) represent the increase in the sum of the squared threshold of the light intensity variation (blue bar) and the corresponding background variation conditions (red or gray).

Figure 14 shows the increase in the mean squared threshold above the no-variation condition. The increase in the squared threshold of the simultaneous variation condition is comparable to the sum of the increase in the squared threshold for the individual variations for both chromatic and achromatic conditions. We used one-way ANOVA to test the hypothesis that the mean increase in squared thresholds for simultaneous variation is equal to the sum of the mean increase in the squared thresholds of light intensity variation and background object reflectance variation conditions. The one-way ANOVA *p*-values are .86 and 0.80 for chromatic and achromatic conditions, respectively. This indicates that the difference between the increase in threshold for the simultaneous variation condition and the sum of the increase thresholds for the individual variations is not statistically significant. The variance of the extrinsic noise calculated for the background variation condition ( $\sigma^{2}=1.00$ , δ=0.00 ) is 0.0015 and the light intensity variation condition ( $\sigma^{2}=0.00$ , δ=0.30 ) is 0.0017. As expected, the variance of the simultaneous variation condition ( $\sigma^{2}=1.00$ , δ=0.30 ), which is 0.0033, is comparable to the sum of individual variances (0.0032).

## Discussion

The visual system maintains a stable representation of object lightness despite variations in the proximal signal due to the scene. We characterized such stability by measuring human observers’ lightness discrimination threshold as a function of variation in the spectra of background objects and in the intensity of light source. We observed that for low variability, the thresholds remained constant, indicating that the thresholds were determined by observers’ intrinsic noise. As variability increased, the extrinsic variation started dominating, increasing thresholds. Using a signal detection theory model, we related the thresholds in the low variability regime to the internal noise and the increase in threshold to the amount of variability in the extrinsic properties. The effects of both types of extrinsic variation, the spectra of background objects, and the intensity of light sources were comparable to the effect of intrinsic noise, indicating strong lightness constancy. Furthermore, the increase in threshold for the simultaneous variation condition was equal to the increase in threshold of the individual variations, indicating that the effect of individual variations add linearly.

Our work builds on previous work on simultaneous variation in illumination and background (Amano & Foster, 2004; Lucassen et al., 2013; Radonjić et al., 2018). In asymmetric color matching experiments with large square shaped Mondrian patterns made of a set of smaller 1° square surfaces, observers show a high degree of constancy for simultaneous changes in the surface position and illuminant as well as illuminant alone (Amano & Foster, 2004). In illumination discrimination experiments with simulated random Mondrian patterns, observers’ thresholds of discrimination were higher for fixed backgrounds surfaces than shuffled backgrounds (Radonjić et al., 2018). These experiments confirm previous work that indicated color appearance depends on the variance of the surround colors (Brown & MacLeod, 1997). Our work aims to quantify the degree of constancy by relating discrimination thresholds to the variance of the surround variability. To match the statistical properties of natural scenes, we use a computational rendering approach to generate images. This approach allows us to generate large scale image datasets with precise control over scene properties, while providing the realism and statistics of natural scenes (Lucassen et al., 2013). Using these datasets, we established that the thresholds of discriminating objects based on their lightness increases with the variance of scene properties.

In experiments involving perceptual judgments, the discrimination threshold quantifies the extent to which an underlying property can change without being detected by an observer. A higher threshold indicates greater perceptual constancy. This interpretation has been used as an evidence of color constancy under illumination changes (Pearce et al., 2014; Radonjić et al., 2016), where observers’ thresholds for detecting change in illumination (an object extrinsic property) was measured, while the surfaces/objects whose appearance was being judged remain fixed. Our experiments differ from this paradigm as we measure the threshold for detecting the change in the object property (its lightness) itself, while object extrinsic properties vary. Our approach measures the property whose constancy is under study (Morimoto & Smithson, 2018). We observe that lightness discrimination thresholds increase with an increase in the amount of variation in object extrinsic properties, suggesting that the visual system compensates for the extrinsic variability, providing a stable representation of the object intrinsic property, a signature of constancy (Singh et al., 2022).

To what extent does the surround affect color perception? A large field of view would allow observers to consider a large number of surfaces and discount large scale properties (Amano & Foster, 2004). However, human observers fixate on a relatively small part of the scene during color judgments (Radonjić et al., 2016). Receptive fields in the human visual cortex, with an extent of ∼1°–2° (Dumoulin & Wandell, 2008; Yoshor et al., 2007), indicate that local interactions play a stronger role in color perception. Non-local changes in chromatic signals affects color appearance only when accompanied simultaneously with background changes adjacent to the target (Wachtler et al., 2001). Additionally, in active, real-world vision, the color awareness falls sharply beyond the center of the field of view, with a third of the observers not noticing any change beyond a 10° field of view (Cohen et al., 2020). Motivated by these observations, we chose our stimuli size as 2° in the visual angle. Although small compared to natural viewing, the images were quite rich in geometry with many different surfaces. The physically realistic rendering included physics-based light–matter interactions and realistic aspects such as shadows and different perspectives of objects and surfaces. Thus, the stimuli allowed for large variance in scene properties and sufficient information in each image to aggregate statistical properties, similar to a larger field of view. Our choice was aimed at capturing the effect of variations that are mediated by neurons in the visual cortex. Extension of our experiments to larger field of view with richer image statistics could provide additional information on the spatial extent of non-local effects (Foster et al., 2006; Hansen et al., 2007).

Our study was limited to spectral variations in the visual scene. We only changed the lightness and chromaticity of the background surfaces. Previous work has shown that thresholds to detect changes in luminance are raised by viewing a field varying in luminance but are unaffected by purely chromatic changes. Similarly, thresholds for detecting bluish-yellowish chromatic changes rise after viewing a field varying in blue-yellow color, but not for a red-green variation (Krauskopf et al., 1982). Moreover, lightness and hue are represented orthogonally in the cortex (Li et al., 2022). We observed that lightness discrimination thresholds of chromatic and achromatic variations were statistically similar. The chromatic aspect of background variation did not affect lightness discrimination, whether coupled with illumination change or not. These observations suggests that the visual system might achieve lightness and chromaticity constancy through independent paths. This hypothesis could be tested by measuring chromaticity discrimination thresholds under chromatic and achromatic background variation.

The increase in lightness discrimination thresholds with a larger spectral variation could be due to lightness assimilation, a phenomenon where the perceived lightness of an object is shifted towards the lightness of neighboring surfaces (Beck, 1966; Hong & Shevell, 2004). The experimental images contained a significant number of background objects of varying sizes and shapes. Their reflectance spectra were sampled independently in every image. As the spectral variations in background objects increase, the assimilation effect would become stronger, producing a confound in judgment and resulting in a higher threshold.

While we limited illumination changes to intensity variation, color constancy depends on the chromaticity of illumination as well as the direction of chromatic change (Aston et al., 2019). Such chromatic bias could be a result of the natural statistics of daylight illumination spectra (Pearce et al., 2014). Illumination discrimination thresholds increase with richer chromatic backgrounds (Radonjić et al., 2018) and color constancy is affected by complexity of the background (Gilchrist et al., 1999). The chromatic aspect of background reflectance could provide cues to compensate for illumination variation (Kraft, 2002). Extending our experimental paradigm to include simultaneous variation in chromatic aspects of illumination and background reflectance spectra could provide a better understanding of the combined role these spectral properties have in color constancy.

Our experimental design allows threshold measurements for different types of extrinsic variations. We observed that the increase in the squared threshold of simultaneous variation of the reflectance spectra of background object and intensity of light sources from the no-variation condition was equal to the sum of the increase in the squared threshold of the individual variations. This indicates that the effects of these variations are independent and add linearly. A similar linear summation rule exists for observers’ attention in images with color gradients and luminance gradients (Engmann et al., 2009). Future work could explore combination rules of other extrinsic properties such as surface position and illuminant (Amano & Foster, 2004), surface and illumination (Craven & Foster, 1992; Ennis & Doerschner, 2019), illumination and depth (Werner, 2006), etc.

The experimental paradigm we have used has previously been used to study contrast detection and contrast noise (Legge et al., 1987; Pelli & Farell, 1999). It was developed to measure the strength of observers’ internal noise and decision efficiency in contrast detection (Lu & Dosher, 1999). One can measure contrast detection thresholds as the extrinsic contrast noise is varied and can use an ideal observer model to obtain the strength of the internal noise and the efficiency of the decision-making process (Baldwin et al., 2016; Lu & Dosher, 1999). Our work follows a similar logic, but there is a major difference. Unlike extrinsic contrast variation in the contrast detection task, the extrinsic variations in our experiments are irrelevant to the task. Furthermore, the dimensions along which different extrinsic properties vary may not be related and may not be directly compararble. Our paradigm allows the comparison of the relative effects of such extrinsic variations.

For the spectral variations studied in this work, the extrinsic variances were within a factor of two compared to intrinsic variance in observers’ representation of lightness. If these variances were equal, one could conclude that the visual system has fully compensated for the extrinsic variation. While the extrinsic variances are larger, they are within a factor of two of the intrinsic variances. This indicates that the visual system provides a large degree of stability in the perceptual representation of lightness and works at near-threshold levels.

## Supplemental Material

sj-pdf-1-ipe-10.1177_20416695241274662 - Supplemental material for Characterization of human lightness discrimination thresholds for independent spectral variations Supplemental material, sj-pdf-1-ipe-10.1177_20416695241274662 for Characterization of human lightness discrimination thresholds for independent spectral variations by Devin Reynolds and Vijay Singh in i-Perception

## Notes

1. The preregistration documents relevant to this work are those for Experiments 6–8. The site also contains preregistrations for previously reported (Experiment 1–3; Singh et al., 2022) and unreported (Experiment 4) work.
