# Supplementary material for: Characterization of human lightness discrimination thresholds for independent spectral variations
Source: Iperception. 2024 Sep 26;15(5):20416695241274662. doi: 10.1177/20416695241274662 (PMC11475098; doi:10.1177/20416695241274662)
Supplement: sj-pdf-1-ipe-10.1177_20416695241274662 - Supplemental material for Characterization of human lightness discrimination thresholds for independent spectral variations [file sj-pdf-1-ipe-10.1177_20416695241274662.pdf]

Supplementary Tables and Figures

Characterization of human lightness discrimination thresholds for independent spectral variations

Devin Reynolds, Vijay Singh.

Department of Physics, North Carolina Agricultural and Technical State University, Greensboro, NC, USA.

Table S1: Thresholds for Background Reflectance Variation Experiment (Preregistered Experiment 6):

Mean threshold (averaged over blocks) $\pm$ SEM of six human observers for nine background variation conditions studied in Background reflectance variation.

Condition	Observer					
	0003	Bagel	Committee	Content	Observer	Revival
$\sigma^2 = 0.00$	0.0221$\pm$0.0010	0.0185$\pm$0.0018	0.0344$\pm$0.0027	0.0223$\pm$ 0.0012	0.0311$\pm$0.0053	0.0251$\pm$0.0023
$\sigma^2 = 0.01$	0.0215$\pm$0.0009	0.0194$\pm$0.0020	0.0386$\pm$0.0103	0.0193$\pm$0.0012	0.0263$\pm$0.0059	0.0262$\pm$0.0048
$\sigma^2 = 0.03$	0.0242$\pm$0.0019	0.0261$\pm$0.0020	0.0285$\pm$0.0029	0.0246$\pm$0.0046	0.0292$\pm$0.0007	0.0282$\pm$0.0016
$\sigma^2 = 0.03$ Achromatic	0.0255$\pm$0.0019	0.0213$\pm$0.0024	0.0343$\pm$0.0055	0.0227$\pm$0.0023	0.0267$\pm$0.0040	0.0263$\pm$0.0016
$\sigma^2 = 0.10$	0.0278$\pm$0.0015	0.0238$\pm$0.0010	0.0284$\pm$0.0017	0.0278$\pm$0.0035	0.0335$\pm$0.0024	0.0281$\pm$0.0013
$\sigma^2 = 0.30$	0.0348$\pm$0.0025	0.0277$\pm$0.0024	0.0344$\pm$0.0020	0.0286$\pm$0.0002	0.0277$\pm$0.0019	0.0301$\pm$0.0038
$\sigma^2 = 0.30$ Achromatic	0.0333$\pm$0.0032	0.0284$\pm$0.0028	0.0319$\pm$0.0047	0.0308$\pm$0.0015	0.0358$\pm$0.0030	0.0287$\pm$0.0022
$\sigma^2 = 1.00$	0.0416$\pm$0.0072	0.0316$\pm$0.0008	0.0379$\pm$0.0024	0.0323$\pm$0.0022	0.0405$\pm$0.0042	0.0360$\pm$0.0055
$\sigma^2 = 1.00$ Achromatic	0.0289$\pm$0.0017	0.0310$\pm$0.0015	0.0391$\pm$0.0029	0.0384$\pm$0.0058	0.0312$\pm$0.0015	0.0322$\pm$0.0009

Table S2. Thresholds for Light Source Intensity Variation Experiment (Preregistered Experiment 7):

Mean threshold (averaged over blocks) $\pm$ SEM of six human observers measured for seven lightness intensity conditions studied in Light source intensity variation. The thresholds of observer Oven were not used in Figure 11.

Condition		Observer				
	0003	Bagel	Oven	Content	Primary	Revival
$\delta = 0.00$	0.0217$\pm$0.0012	0.0181$\pm$0.0001	0.0520$\pm$0.0114	0.0208$\pm$0.0014	0.0329$\pm$0.0061	0.0372$\pm$0.0008
$\delta = 0.05$	0.0228$\pm$0.0018	0.0229$\pm$0.0018	0.0580$\pm$0.0064	0.0207$\pm$0.0007	0.0346$\pm$0.0042	0.0364$\pm$0.0013
$\delta = 0.10$	0.0275$\pm$0.0024	0.0217$\pm$0.0009	0.0325$\pm$0.0022	0.0242$\pm$0.0040	0.0343$\pm$0.0013	0.0376$\pm$0.0072
$\delta = 0.15$	0.0316$\pm$0.0009	0.0238$\pm$0.0011	0.0333$\pm$0.0019	0.0323$\pm$0.0032	0.0345$\pm$0.0042	0.0326$\pm$0.0002
$\delta = 0.20$	0.0447$\pm$0.0100	0.0381$\pm$0.0046	0.0493$\pm$0.0120	0.0276$\pm$0.0016	0.0423$\pm$0.0050	0.0392$\pm$0.0034
$\delta = 0.25$	0.0433$\pm$0.0052	0.0393$\pm$0.0062	0.0461$\pm$0.0060	0.0308$\pm$0.0023	0.0532$\pm$0.0083	0.0387$\pm$0.0025
$\delta = 0.30$	0.0404$\pm$0.0018	0.0429$\pm$0.0033	0.0580$\pm$0.0061	0.0347$\pm$0.0014	0.0465$\pm$0.0047	0.0421$\pm$0.0042

Table S3. Thresholds for Simultaneous Variation Experiment (Preregistered Experiment 8):
Mean threshold (averaged over blocks) ± SEM of six human observers measured for six conditions studied in preregistered experiment 8.

Condition	Observer					
	0003	Bagel	Oven	Content	Manos	Revival
No Variation	0.0261±0.0022	0.0227±0.0019	0.0383±0.0066	0.0246±0.0004	0.0258±0.0036	0.0366±0.0085
Background Variation Chromatic	0.0414±0.0036	0.0340±0.0058	0.0498±0.0050	0.0392±0.0083	0.0306±0.0013	0.0383±0.0033
Background Variation Achromatic	0.0394±0.0027	0.0319±0.0015	0.0683±0.0048	0.0427±0.0074	0.0435±0.0071	0.0389±0.0010
Light Intensity Variation	0.0464±0.0027	0.0656±0.0208	0.0592±0.0091	0.0412±0.0021	0.0464±0.0046	0.0474±0.0069
Simultaneous Variation Chromatic	0.0635±0.0092	0.0536±0.0014	0.0639±0.0106	0.0437±0.0011	0.0768±0.0085	0.0528±0.0037
Simultaneous Variation Achromatic	0.0648±0.0103	0.0540±0.0017	0.0826±0.0166	0.0478±0.0049	0.0749±0.0082	0.0561±0.0028

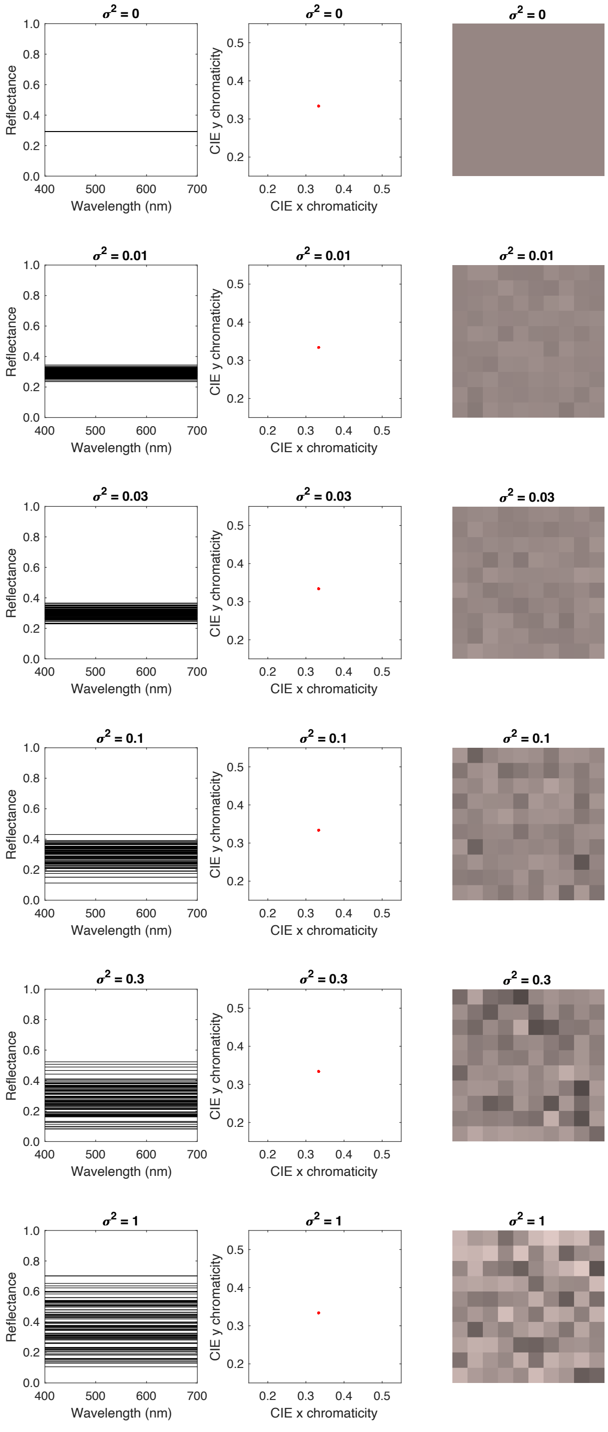

Figure S1: Statistical model of surface reflectance: Same as Figure 4 for achromatic condition.

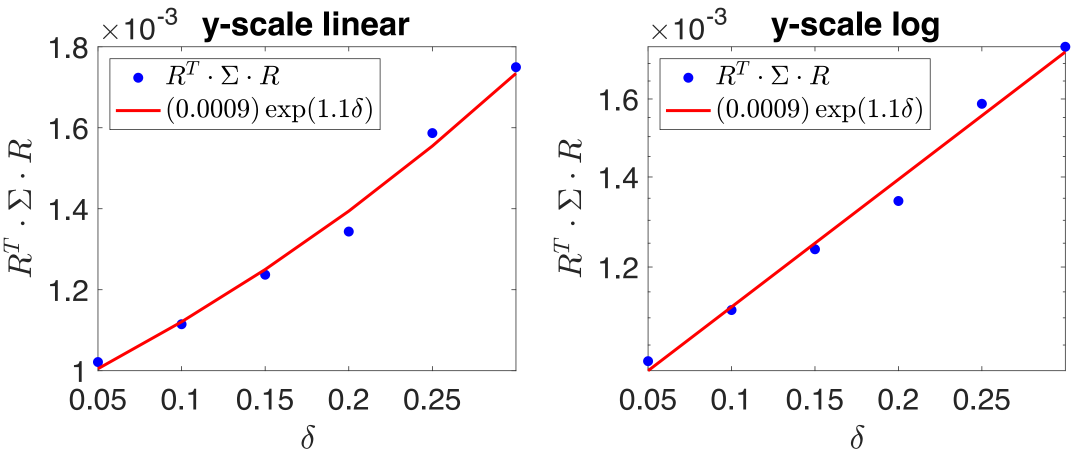

Figure S2: Estimation of extrinsic noise for *light source intensity variation* experiment: Plot of the variance ($R^T \Sigma R$) as a function of the range parameter δ on a linear (left panel) and logarithmic (right panel) scale. We fit the function with an exponential of the form $A * \exp{(B \cdot \delta)}$. The variance in the extrinsic noise is estimated as the value of the fit at $\delta = 1$.

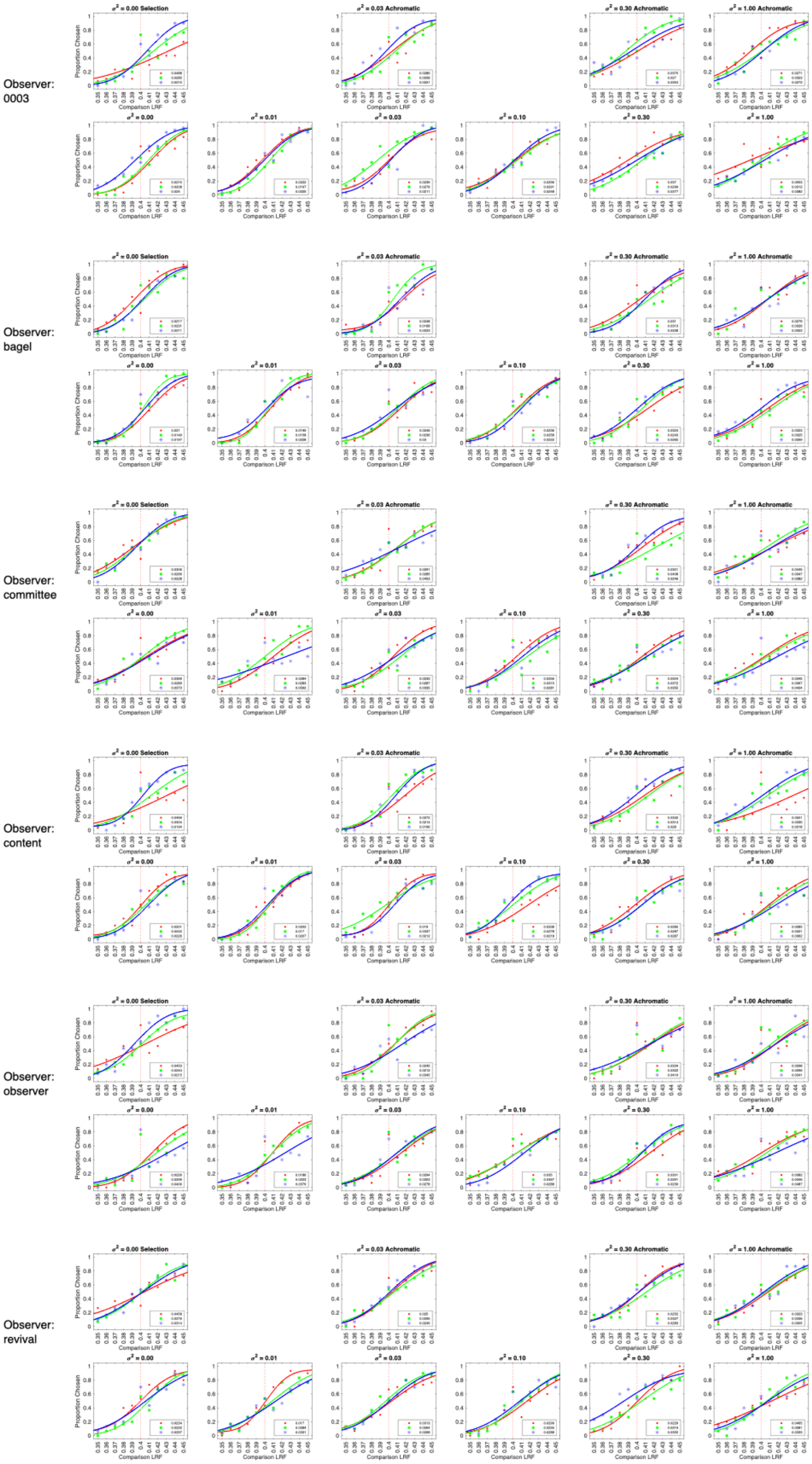

Figure S3: Psychometric functions for all observers for background reflectance variation experiment. Same as Figure 8, for all observers retained in the *background reflectance variation* experiment.

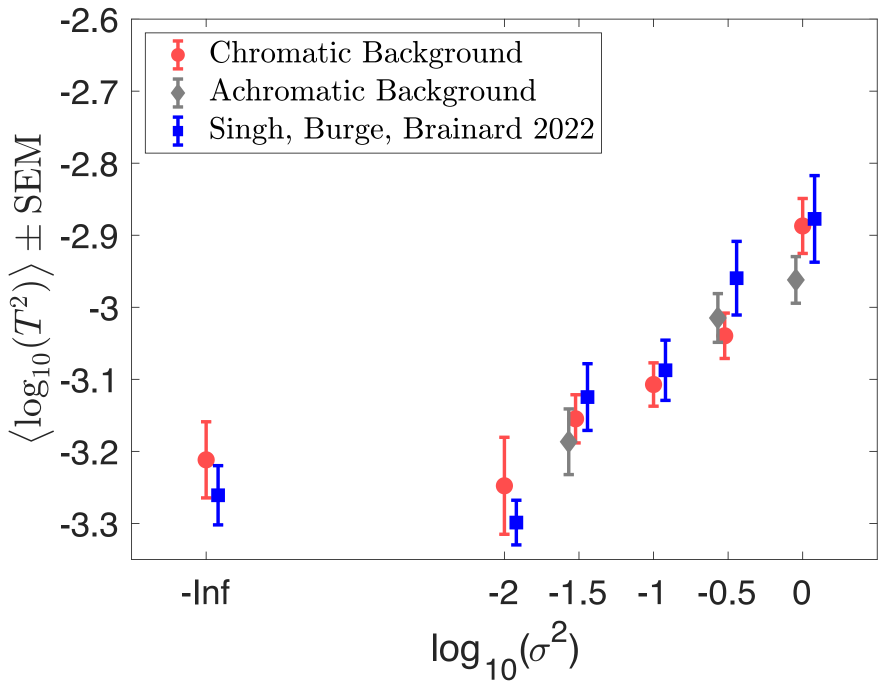

Figure S4: Comparison with Singh, Burge, Brainard 2022. Lightness discrimination thresholds for background variation condition measured in *background reflectance variation* experiment and previously reported data from Singh, Burge, Brainard (2022). The previous experiment only had chromatic conditions and made three threshold measurements for each condition for 4 naïve observers. In this work, *background reflectance variation* experiment had both chromatic and achromatic conditions and measured thresholds for six observers. The experiments were otherwise the same.

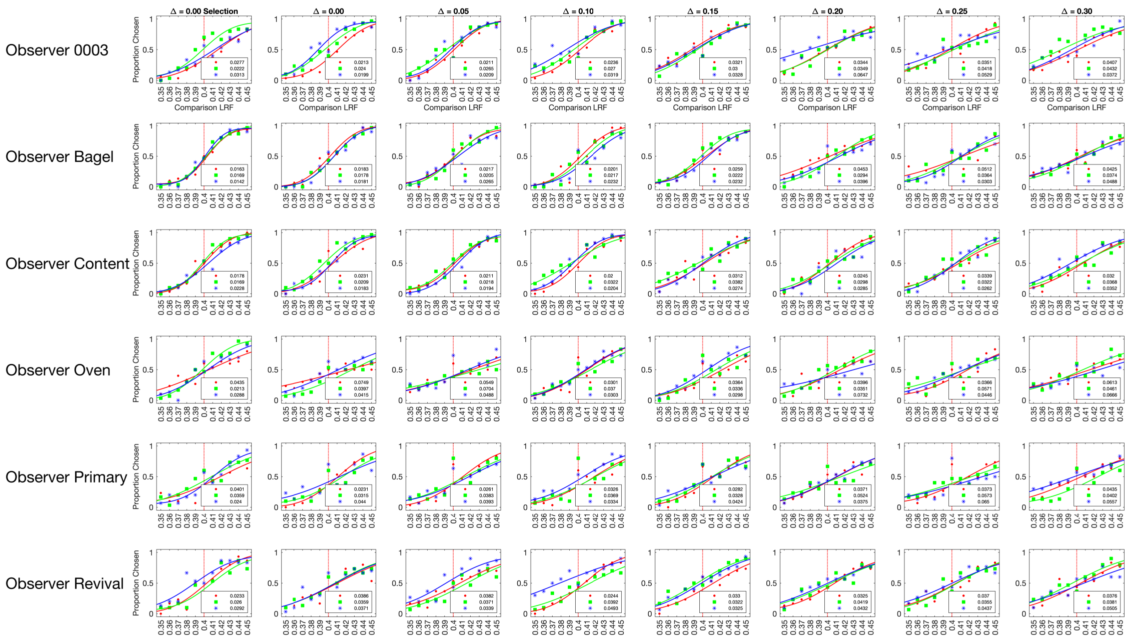

Figure S5: Psychometric functions for all observers for light intensity variation experiment. Same as Figure 10, for all observers retained in the *light source intensity variation* experiment.

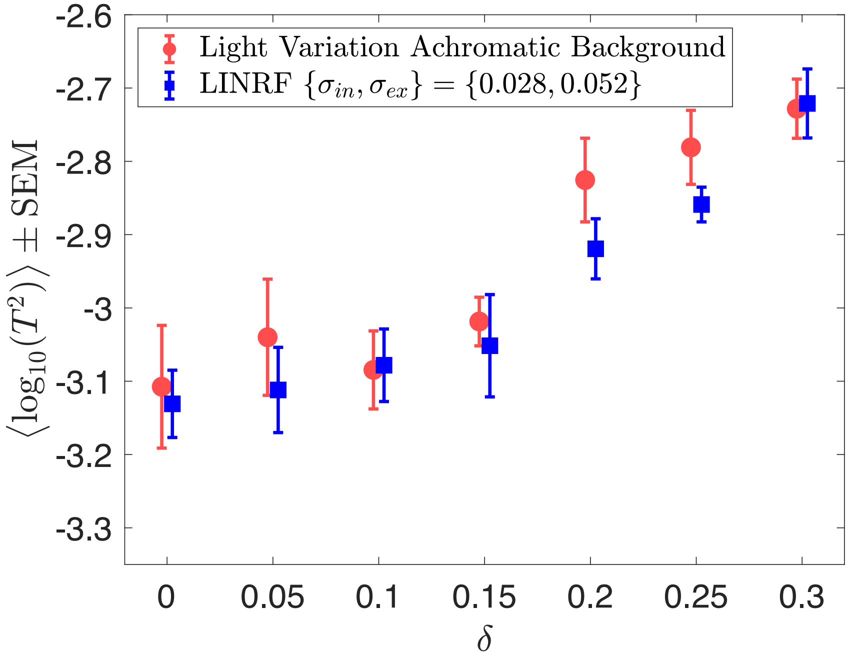

Figure S6: Same as Figure 11, for all six observers retained in the *light source intensity variation* experiment. The parameters for the LINRF model are the same as in Figure 11.

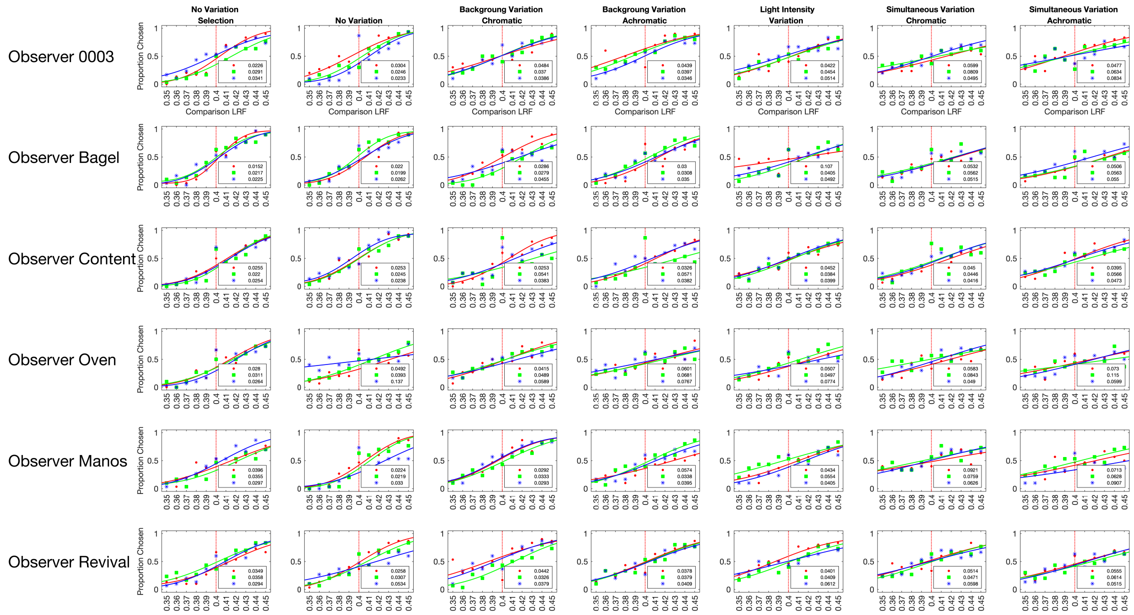

Figure S7: Psychometric functions for all observers for Simultaneous variation experiment. Similar to Figure 12, for all observers retained in the *simultaneous variation* experiment.

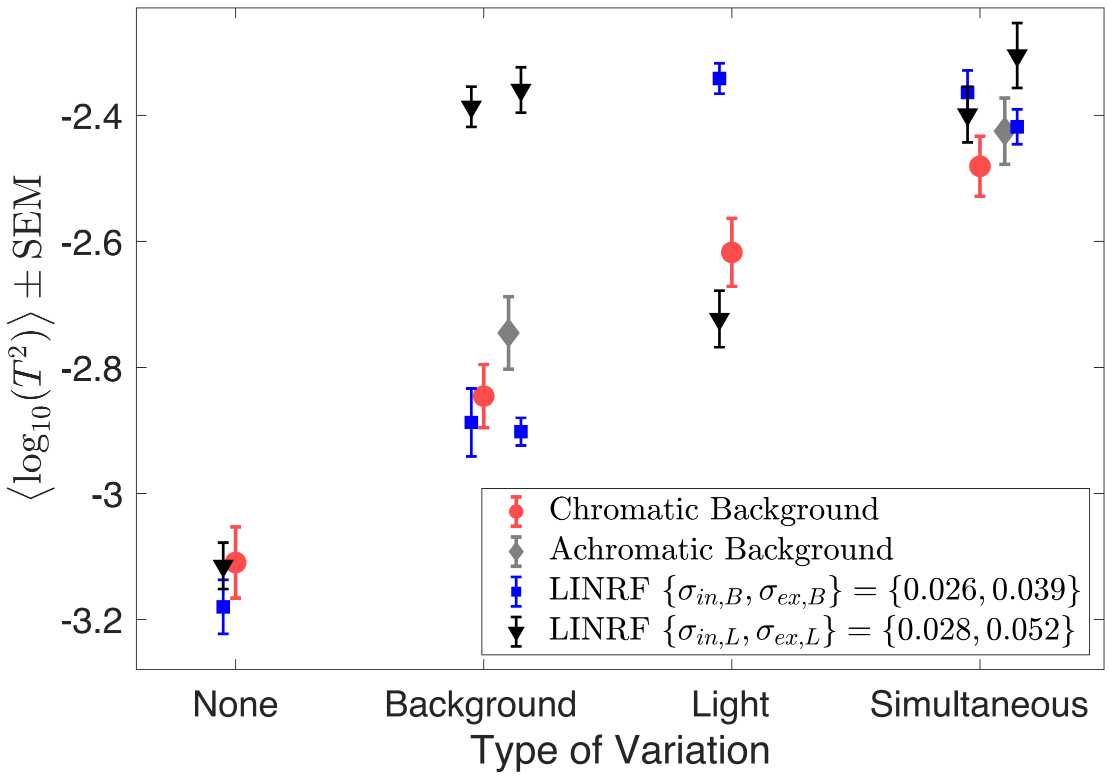

Figure S8: Same as Figure 13, but the thresholds of the linear receptive field (LINRF) model were estimated using the same set of parameters for all six conditions studied in Simultaneous variation experiment. Blue square markers show log squared thresholds estimated using the parameters of the Background reflectance variation experiment (Figure 9). Black triangular markers show log-squared thresholds estimated using the parameters of the Light intensity variation condition (Figure 11). The blue error bars show +/- 1 standard deviation estimated over 10 independent estimates of the LINRF model parameters. The parameters of the *background reflectance variation* condition (blue squares) predict the thresholds of the no-variation condition, the background reflectance variation condition, and the simultaneous variation condition quite well, but fail to predict the threshold of the light source intensity variation condition. Similarly, the parameters of the *light source intensity variation* experiment (black triangles) predict the thresholds of the no-variation condition, the light source intensity variation condition, and the simultaneous variation condition quite well, but fail to predict the threshold of the background variation condition. This could possibly be because the observers in the three experiments were different. Future work would aim at studying these conditions using the same set of observers.
